# Spatial Heterogeneity of Digital HIV/AIDS Stigma in China: Cross-Sectional Ecological Study

**DOI:** 10.2196/93007

**Published:** 2026-10-09

**Authors:** Pu Zhang, Yiliang Li, Zheng Wei, Na Jiang, Pan Hui

**Affiliations:** 1 The Hong Kong University of Science and Technology (Guangzhou), 1st, Duxue Road, Guangzhou, Guangdong, 511453, China, 86 19575360103; 2 Wuhan University, Wuhan, Hubei, China; 3 The Hong Kong University of Science and Technology, Hong Kong, China (Hong Kong); 4 Guangdong Provincial Key Laboratory of Smart and Safety City Planning and Monitoring, Guangzhou, Guangdong, China; 5 University of Helsinki, Uusimaa, Helsinki, Finland

**Keywords:** HIV/AIDS, stigma, infoveillance, short-video platforms, large language models, spatial analysis, digital health

## Abstract

**Background:**

HIV/AIDS stigma remains a major barrier to public health intervention and social inclusion. In China, short-video platforms, such as Douyin, have become important spaces where people living with HIV/AIDS share daily-life narratives and receive public responses. However, little is known about how public responses to these narratives vary geographically, or how regional sociodemographic and epidemiological contexts are associated with digital stigma and support.

**Objective:**

This study aimed to examine the thematic structure, spatial heterogeneity, and macro-level correlates of public responses to daily-life narratives shared by people living with HIV/AIDS on Douyin in China, with a focus on spatially varying correlational patterns of digital HIV/AIDS stigma.

**Methods:**

We constructed a corpus of 174,394 public comments from Douyin videos created by people living with HIV/AIDS. DeepSeek R1 671B was used to classify comments into 9 substantive themes and 1 residual “others” category. Provincial topic indices were calculated by normalizing thematic comment counts by mobile internet access users. We then combined the least absolute shrinkage and selection operator feature selection, ordinary least squares, and spatial econometric models, residual spatial autocorrelation diagnostics, false discovery rate correction, and Shapley Additive Explanations (SHAP)–geographic information system (GIS) interpretation to examine global associations and localized variable contributions across 31 provincial-level units in mainland China.

**Results:**

Moral judgment and attribution of blame were the most prevalent substantive theme (41,663/174,394, 23.89%), followed by disease knowledge and transmission discussion (23,344/174,394, 13.39%), and support, encouragement, and humanitarian care (18,101/174,394, 10.38%). Spatial analysis showed pronounced geographic heterogeneity in both stigma- and support-oriented public responses. HIV incidence was positively associated with disease knowledge, social impact discussion, moral judgment, and support, while gender ratio was significantly associated with moral judgment and related evaluative themes. SHAP-GIS further revealed stronger positive contributions of HIV incidence to moralizing discourse in several southwestern provinces and a marked north-south pattern in the contribution of gender ratio.

**Conclusions:**

Digital public responses to daily-life narratives shared by people living with HIV/AIDS in China are geographically uneven and associated with place-based sociodemographic and epidemiological contexts.

## Introduction

### Background

AIDS, caused by HIV, remains a formidable challenge at the intersection of public health and societal behavior. By 2024, approximately 40.8 million people were living with HIV globally, with 630,000 AIDS-related deaths annually, underscoring a persistent crisis that biomedical advances alone cannot resolve [1]. In mainland China, the number of people living with HIV/AIDS exceeds 1 million, with rising diagnosis and mortality rates [2]. These epidemiological trends are inextricably linked to sociobehavioral determinants (eg, social stigma, health literacy deficits, and psychological barriers [3]), which not only undermine the well-being of people living with HIV/AIDS but also obstruct systemic efforts to achieve the “95-95-95” public health targets [4,5].

The rapid evolution of computer-mediated communication has fundamentally reshaped how the public consumes and responds to health-related narratives. Short-form video platforms, exemplified by Douyin (the Chinese version of TikTok), have transitioned from entertainment hubs into primary sociotechnical landscapes for information exchange among hundreds of millions of users [6,7]. The short-video format, characterized by user-generated videos typically under 60 seconds with algorithm-driven content distribution, constitutes the primary medium through which these interactions occur on Douyin [8]. For people living with HIV/AIDS, these platforms offer a unique digital affordance for self-disclosure, peer support, and the building of psychological resilience [9]. However, the digital environment is a double-edged sword; the anonymity and high-speed virality of Douyin can facilitate online disinhibition, leading to the rapid proliferation of stigmatizing content and the reinforcement of negative stereotypes [10,11].

Despite the proliferation of HIV/AIDS-related user-generated content, a significant research gap remains regarding the digital determinants of health [12,13], particularly in relation to how online expressions of HIV/AIDS stigma and support are shaped by place-based contexts. In this study, digital HIV/AIDS stigma refers to online expressions of social disapproval, blame attribution, and negative stereotyping toward people living with HIV/AIDS, whereas digital support refers to expressions of empathy, encouragement, and humanitarian care. Specifically, it remains unclear how macro-environmental correlates modulate online behavior [14]. While individual-level psychology is well documented, we lack systematic evidence on how broader regional socioeconomic and epidemiological contexts influence the way netizens interpret and react to narratives of people living with HIV/AIDS [15-17]. Drawing on the concept of infoveillance, we regard comments on Douyin videos created by people living with HIV/AIDS as observable digital traces of public responses to HIV/AIDS narratives [18]. These responses range from moral condemnation, fear-based distancing, and blame attribution to informational exchange and humanitarian support. Understanding this spatial nonstationarity in human behavior, where exposure to similar digital content may elicit divergent responses depending on the user’s geographic and social substrate, is essential for advancing theories of risk perception and digital health governance [5,19]. Detailed examples and coding criteria, including the distinction between stigmatizing comments and negative in tone but factually accurate statements, are provided in Multimedia Appendix 1.

To address these gaps, this study uses a large-scale dataset of 174,394 public comments collected from Douyin. We integrate large language models (LLMs) for automated thematic classification with spatial econometric modeling and machine learning interpretability (Shapley Additive Explanations [SHAP]) to decode the complex relationship between regional context and online health attitudes. Specifically, this study is guided by the following 4 research questions (RQs):

- RQ1: What are the primary thematic dimensions and behavioral patterns in the public’s digital discourse regarding the daily-life narratives of people living with HIV/AIDS on short-video platforms?
- RQ2: How is the intensity of these digital themes distributed geographically across China’s provinces, and are there significant spatial clusters of stigma or support?
- RQ3: To what extent are provincial socioeconomic (eg, gross domestic product [GDP] and gender ratio) and epidemiological (eg, HIV incidence) factors associated with the global prevalence of these digital health attitudes?
- RQ4: How do the model-estimated relationships of these macro-level correlates exhibit spatial nonstationarity, and which specific regional contexts catalyze or inhibit the digital manifestation of HIV stigma?

By bridging computational linguistics with behavioral geography, this study provides insights into how digital technology and local environments intersect to shape human reactions to one of the world’s most stigmatized health conditions. Our findings contribute to the vision of “zero discrimination” advocated by the Joint United Nations Programme on HIV/AIDS (UNAIDS) and offer an approach for future research on computer-mediated social support and digital health governance.

### Theoretical Framework: Sociohealth Correlates of Place

In health geography, “place” is conceptualized as a complex intersection of social, demographic, and epidemiological contexts that collectively modulate public health perceptions. We draw on the socioecological model to organize our analytical framework. The socioecological model posits that health-related behaviors and attitudes are shaped by multiple interacting levels: intrapersonal, interpersonal, community, and societal [20]. While our data do not permit individual-level analysis, we apply the socioecological model’s core insight at the macro-level by examining how provincial-level contextual factors, including demographic, epidemiological, and socioeconomic conditions, are associated with regional patterns in digital discourse. Specifically, the socioecological model guides our selection of variables by identifying 3 contextual domains that prior literature has linked to health-related attitudes: population composition (demographic substrate), disease burden (epidemiological context), and material conditions (socioeconomic foundation).

### The Demographic and Cultural Substrate

The demographic environment serves as the cultural and social substrate for empathy and moral judgment, as it defines the normative background of a community [21]. Previous research has established that social environments influenced by different demographic structures can exhibit different health-related communication styles [22-24]. Within the socioecological model, this dimension corresponds to the community-level normative environment that shapes interpersonal attitudes. Gender ratio reflects gendered socialization patterns within a province, which are associated with prevailing moral norms and communication styles regarding sensitive health topics. Life expectancy serves as a proxy for overall health resilience and social stability, indicating the public’s baseline health security. Average schooling years, as a primary determinant of health literacy, acts as a cognitive resource that may reduce the proliferation of irrational stigma [23].

### The Epidemiological Context

The epidemiological context draws on the logic of contact theory, which suggests that the visibility and prevalence of a disease within a place may shape public attitudes through increased salience and familiarity [25,26]. We do not equate provincial HIV incidence with direct interpersonal contact; rather, we conceptualize these indicators as structural background factors that condition the discursive environment. Within the socioecological model, this dimension represents the community-level disease burden that shapes risk perception and threat appraisal. HIV incidence rate reflects the current risk and epidemiological salience within a province; high incidence may be associated with either increased social awareness or defensive stigma as a psychological distancing mechanism. HIV mortality rate captures the perceived severity of the disease, which often sustains fear-based stigmas and may inhibit humanitarian support.

### The Socioeconomic Foundation

The socioeconomic foundation provides the material basis for digital literacy and the diversification of values in the digital public sphere. Economic development influences whether digital discourse is dominated by survival-based anxieties or postmaterialist empathy [27,28]. Within the socioecological model, this dimension captures the community-level resource environment that enables or constrains health-related communication. GDP per capita, as a measure of economic development, is associated with greater exposure to pluralistic health perspectives and higher social capital. Consumption expenditure per capita, representing actual living standards, determines the disposable social and cognitive resources available for digital engagement.

## Methods

### Overview

This section delineates the integrated methodological framework designed to investigate the sociospatial correlates of digital HIV/AIDS discourse in China. This study used a cross-sectional ecological design combining provincial-level socioeconomic and epidemiological data with social media comment data. As illustrated in Figure 1, our approach follows a symmetric convergence model that bridges macro-environmental indicators with micro-level digital behaviors. The methodology is structured into 3 primary phases:

1. Sociohealth data and corpus preparation: We systematically acquired provincial socioeconomic and epidemiological indicators and constructed a large-scale digital corpus of 174,394 comments from Douyin.
2. LLM-based behavioral classification: Using the DeepSeek R1 large language model, we categorized the discourse into 9 thematic dimensions to quantify public perceptions across mainland China.
3. Spatial and interpretable mMachine learning analysis: We integrated spatial econometric modeling to identify global associations between macro-level contextual factors and digital discourse outcomes and used SHAP-geographic information system (GIS) to decompose these associations into localized marginal contributions, thereby accounting for spatial nonstationarity.

**Figure 1.**
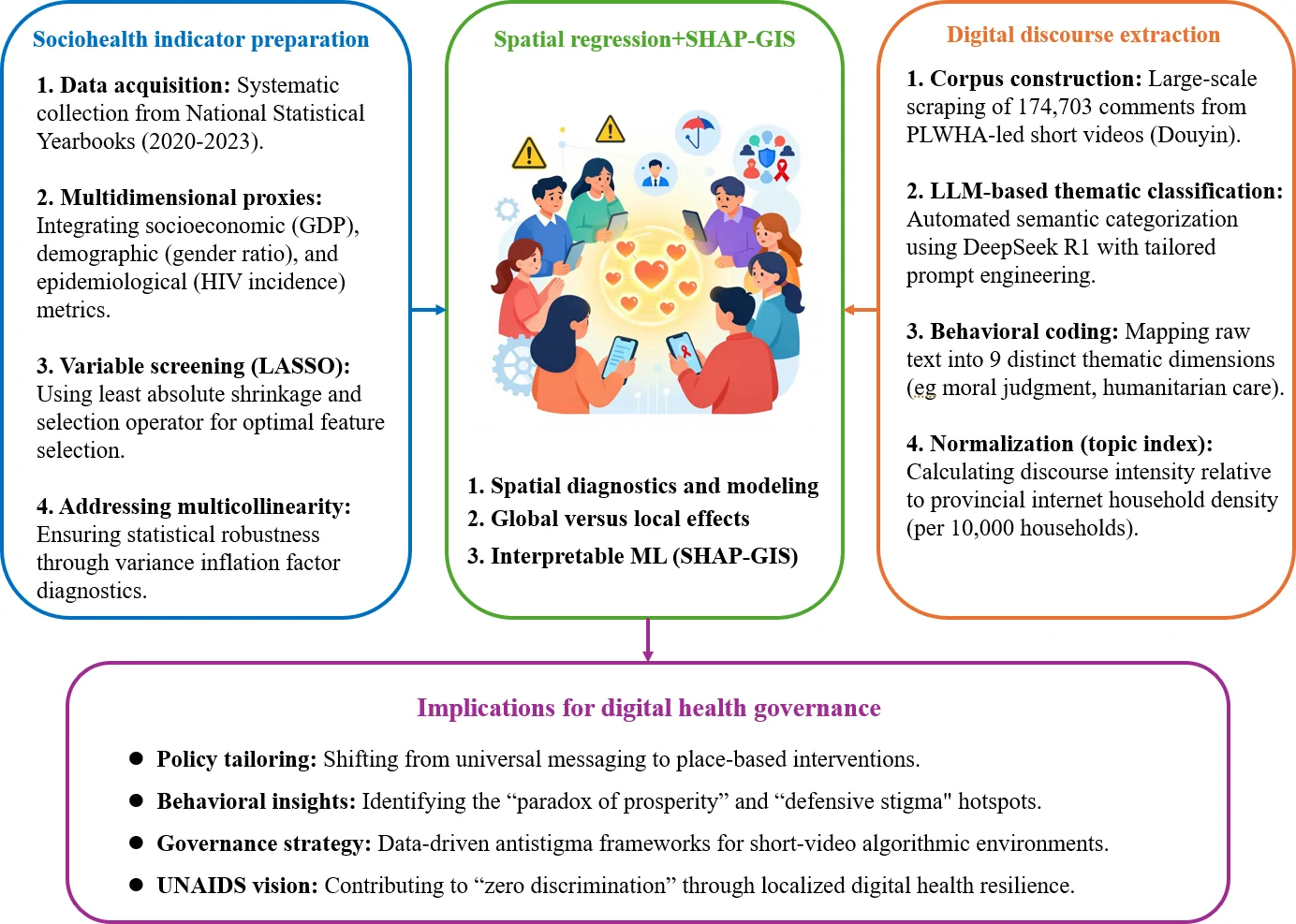
An integrated computational pipeline for decoding digital HIV/AIDS discourse. GDP: gross domestic product; GIS: geographic information system; LASSO: least absolute shrinkage and selection operator; LLM: large language model; ML: machine learning; SHAP: Shapley Additive Explanations; UNAIDS: Joint United Nations Programme on HIV/AIDS.

Subsequent subsections provide detailed specifications for the technical implementation and validation of each component.

### Data Preparation

The data collection process began on May 18, 2025, with a keyword search on Douyin using the term “抗艾日常” (roughly translated as “my anti-AIDS life” or “life with HIV/AIDS”). This keyword was selected following a pilot search (May 10‐15, 2025), which identified it as the most active hashtag for daily-life narratives of people living with HIV/AIDS on Douyin, capturing first-person content directly relevant to our research question. Importantly, Douyin uses a fuzzy search mechanism for content retrieval, meaning that a single keyword query captures videos containing variations or related expressions of the hashtag, ensuring comprehensive coverage within the thematic domain. The search initially yielded 311 video results. Two independent reviewers screened the titles and descriptions of all 311 videos. The inclusion criteria were (1) people living with HIV/AIDS sharing daily-life experiences, (2) more than 1000 comments, and (3) Chinese-language content. Interreviewer agreement was 94.5% (Cohen *κ*=0.89), with disagreements resolved through consultation with a third reviewer. We acknowledge that this keyword selection biases our sample toward users who actively engage with daily-life content shared by people living with HIV/AIDS, potentially excluding those who engage only with news-based or educational HIV/AIDS content; this is discussed as a limitation in the *Discussion* section.

After screening, 91 videos were identified as highly relevant to the research focus (a representative video and typical comments are shown in Figure 2), specifically those in which people living with HIV/AIDS shared their daily life experiences and were also relatively popular (ie, having more than 1000 comments). Subsequently, web scraping techniques were used to collect all publicly available comments under these 91 selected videos, resulting in a dataset comprising 291,264 individual comments.

**Figure 2.**
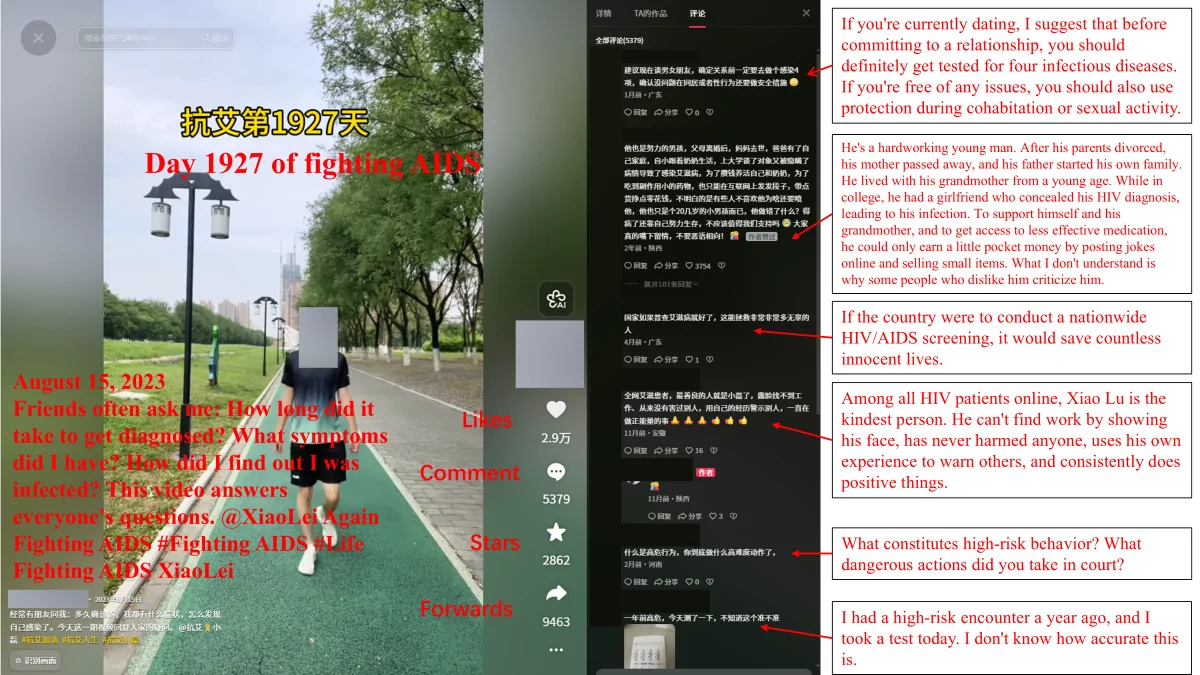
Representative videos and typical comments.

Next, the initial comment data underwent a rigorous cleaning process [29,30]. This multistep procedure included (1) converting emojis into their corresponding Chinese character descriptions (eg, “[Smile]” for a smiling emoji) to facilitate textual analysis; (2) removing all instances of “@username” mentions; (3) deleting other nonessential characters, such as special symbols or excessive punctuation that did not contribute to the comment’s meaning; (4) filtering out comments containing fewer than 6 Chinese characters, as they were deemed to possess insufficient informational content for meaningful analysis; and (5) excluding comments originating from IP addresses outside mainland China to focus the analysis on perceptions within the Chinese sociocultural context. Following these data preparation steps, the final dataset consisted of 174,394 valid comments for the analysis. The specific distribution of comment data is shown in Figure 3. All exclusion criteria were predefined based on observable comment characteristics rather than outcome-related variables.

All 31 provincial-level administrative regions in mainland China were included. For social media data, all publicly available comments under the selected videos during the study period were collected without additional sampling.

**Figure 3.**
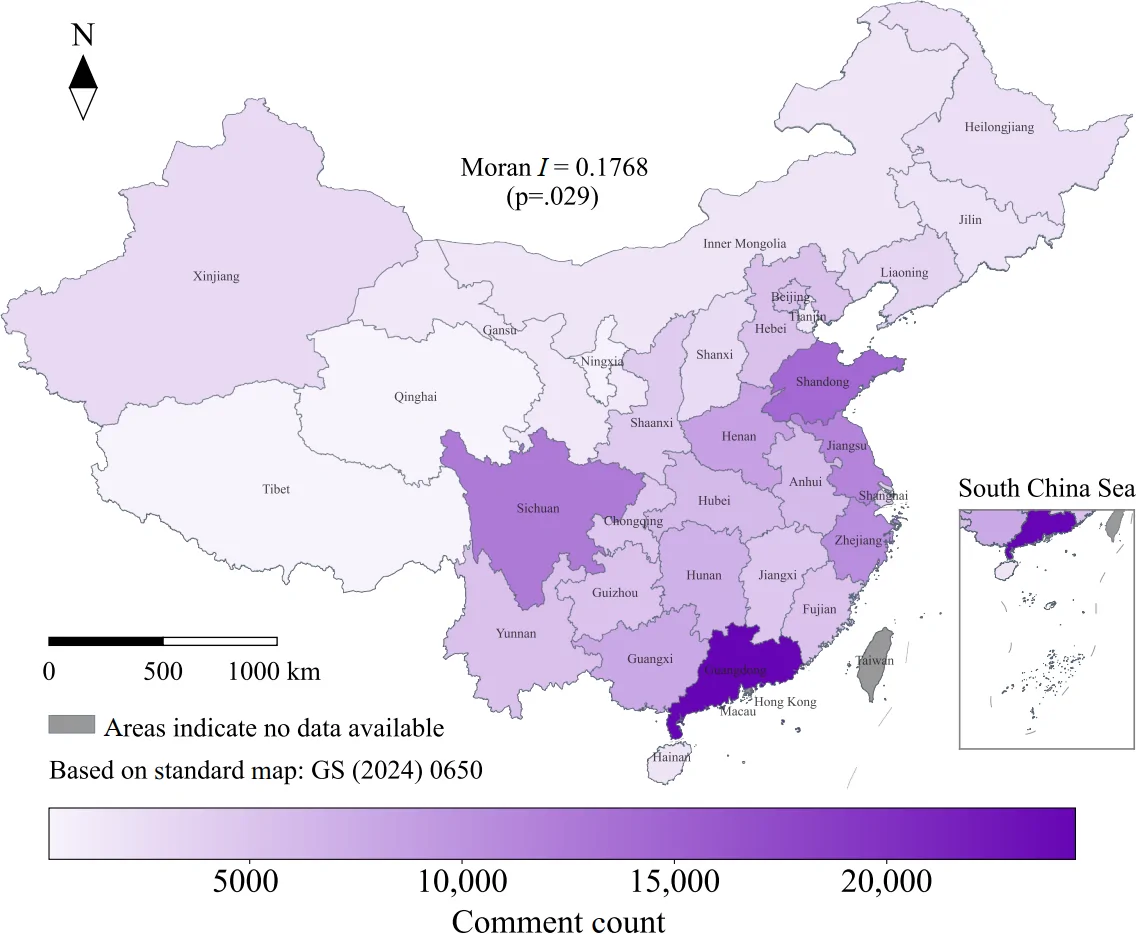
Distribution of raw comments across 31 provincial-level units in mainland China. All maps are based on the standard map with approval GS (2024) 0650 from the Ministry of Natural Resources of China. Darker shades indicate higher prevalence, while lighter shades indicate lower prevalence. The local Moran *I* statistic indicates significant spatial clustering.

### Text Classification

To comprehensively analyze the rich discussion features within the comments and categorize the diverse public perceptions, we adopted an analytical approach based on LLM-driven few-shot learning. Existing research has demonstrated the high accuracy and efficacy of LLM-driven few-shot learning paradigms for nuanced thematic classification of large-scale social media text data [31,32]. Given these established capabilities, we selected the *DeepSeek R1* API to facilitate the thematic analysis for this study [33]. This approach was deemed most suitable for systematically and accurately processing our extensive and complex dataset of user comments [34]. The prompt and core Python code used in this study can be found on GitHub [35].

The development of the thematic category framework was initiated by randomly selecting 5000 comments from the cleaned dataset. Adhering to open coding principles [36], the research team iteratively analyzed this subset to identify potential themes. The specific process for identifying the topic is shown in Figure 4.

**Figure 4.**
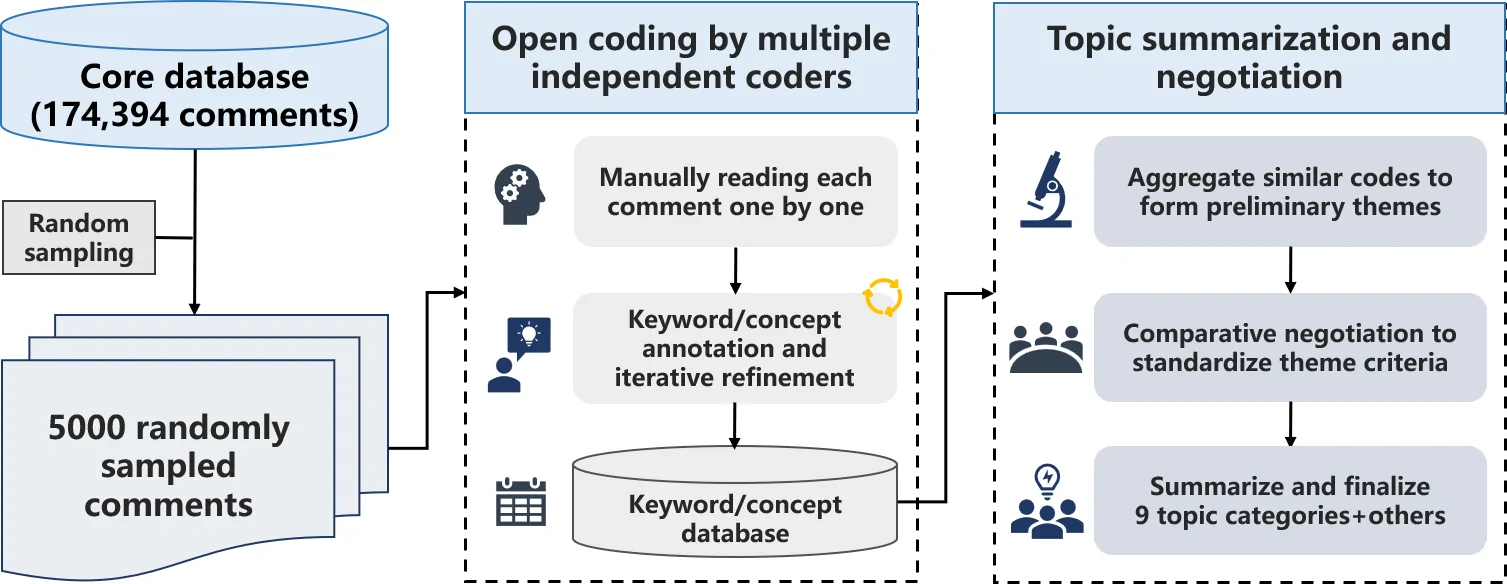
Workflow for identifying topics in accordance with open coding principles.

Through systematic analysis and synthesis, this study identified 10 main thematic categories, each with corresponding definitions, as follows:

1. *Disease knowledge and transmission discussion*: This category focuses on medical knowledge of HIV/AIDS, including transmission patterns, incubation period, symptoms, diagnostic methods, prevention measures, and treatment advances. Comments in this category are characterized by inquiries, information sharing, popular science education, or myth-busting. For example, some users asked, “How do I know if I have AIDS?”
2. *Humor, banter, and ironic expression*: Comments in this category use humor, banter, exaggeration, satire, or playful language to address HIV/AIDS-related topics. Such expressions may serve as a means of emotional regulation but may also contain sarcasm or inappropriate connotations. For example, “Even AIDS can be cured. When will my gout be cured?”
3. *Information sources and credibility discussion*: This category focuses on the accuracy and reliability of medical information, news reports, and expert opinions. Comments typically involve assessments or doubts about the authenticity, authority, and timeliness of the information. For example, “Even the world’s top medical teams can’t handle this; you’re overthinking it.”
4. *Moral judgment and attribution of blame*: Comments in this category involve a moral assessment of the causes of infection or patient behavior, often stigmatizing the disease by attributing it to specific behaviors or groups and expressing condemnation, discrimination, or prejudice. For example, “Except for those infected by blood transfusion, others do not deserve sympathy.”
5. *Personal experience and current situation sharing*: This category includes users sharing their own infection-related experiences, symptoms, treatment processes, psychological states, or describing individuals’ health status and life details after infection. For example, “Just treat it as a chronic disease, take medication on time. Now my viral load is undetectable, and I’m working hard to provide a better life for my family.”
6. *Risk underestimation and cognitive bias*: Comments in this category reflect an underestimation of the risk of HIV infection or the severity of the disease or display misconceptions about viral transmission. For example, “Compared to cancer and cardiovascular diseases, AIDS is not a disease.”
7. *Social impact and public issue discussion*: This category addresses the association of HIV/AIDS at both individual and societal levels, including social relationships, family, employment, mental health, discrimination, policy recommendations, public health, and legal responsibilities. For example, “Can this kind of situation be prosecuted and sentenced?”
8. *Support, encouragement, and humanitarian care*: Comments in this category express sympathy, understanding, support, and encouragement for people living with HIV/AIDS, call for the elimination of discrimination, respect for privacy, or share positive coping attitudes. For example, “Child, don’t listen to what they say. The words of others are meaningless. If you are sick, get treatment and take care of yourself.”
9. *Worry, fear, and anxiety emotions*: This category reflects users’ concerns and anxieties about infection risks, the disease itself, or social discrimination. For example, “Now I think it’s better to stay single, I’m afraid of falling in love.”
10. *Others*: This category includes comments that cannot be classified into any of the aforementioned categories, such as content unrelated to HIV/AIDS and people living with HIV/AIDS, meaningless characters, or scattered remarks with indeterminate intent. For example, “This can’t be downloaded, why?”

These predefined themes were then used to guide the DeepSeek R1 671B model in conducting a few-shot classification of the entire 174,394 comments. The model was deployed using the open-source version on our university’s high-performance computing center, without any modification or quantization. The few-shot prompts were designed with clear task definitions, category descriptions accompanied by representative examples, and explicit single-label classification instructions to ensure consistency. All classification prompts and instructions were provided in Chinese to align with the language of the comments. For each comment, the model was instructed to assign exactly one best-fitting category from the predefined theme list, with no option for multiple assignments or uncertain classification. Comments that could not be confidently assigned to any substantive theme were classified into a residual “Others” category, which was treated as noise and excluded from further thematic analysis. To ensure reproducibility, we set the model temperature to 0. The full classification prompt and core code are provided on GitHub [35].

To validate the LLM’s classification accuracy, a stratified random sample was constructed by selecting 100 comments per category (1000 total) from the classified corpus, ensuring adequate representation of lower-frequency themes. This validation sample was manually coded by 2 independent coders blind to the LLM labels, with intercoder agreement. Disagreements were resolved through discussion. The LLM’s classification for this subset was compared against human-coded labels. The model achieved 95.20% overall accuracy (48/1000 errors) with a Cohen κ of 0.9467, indicating excellent agreement beyond chance [37]. Each thematic category accuracy’s is detailed in Table 1, and the full confusion matrix is provided in Figure SA-1 in Multimedia Appendix 2. These results confirm that the LLM-based classification effectively covers the breadth and complexity of the discussion content in the comments.

**Table 1.** Classification performance assessment based on stratified sampling (100 per category).

Topic categories	Precision^a^^,^^b^	Recall^a^^,^^b^	*F*_1_-score^a^^,^^b^
Disease knowledge and transmission discussion	0.970	0.990	0.980
Humor, banter, and ironic expression	0.980	0.778	0.867
Information sources and credibility discussion	0.960	1.000	0.980
Moral judgment and attribution of blame	0.930	0.959	0.944
Personal experience and current situation sharing	0.900	0.918	0.909
Risk underestimation and cognitive bias	0.980	1.000	0.990
Social impact and public issue discussion	0.940	0.979	0.959
Support, encouragement, and humanitarian care	0.990	0.990	0.990
Worry, fear, and anxiety emotions	0.980	1.000	0.990
Others	0.890	0.957	0.922

a Macro average: precision=0.952; recall=0.957; *F*_1_-score=0.953.

b Weighted average: precision=0.953; recall=0.952; *F*_1_-score=0.951.

### Influencing Factor Analysis

To analyze the socioeconomic correlates of localized HIV/AIDS discourse, we implemented a multistage quantitative framework. This approach transitioned from variable selection and global regression to spatial diagnostics and advanced local interpretation.

### Dependent Variable: The Normalized Topic Index

The primary dependent variable in this study is the Normalized Topic Index, representing the intensity of public engagement with specific HIV/AIDS-related themes across the 31 provinces. To ensure that our analysis reflects regional social attitudes rather than mere differences in digital infrastructure or population size, we normalized the absolute discussion counts (ie, comment volume) for each province [38]. The normalization was performed by dividing the total comment count for a specific topic by the number of mobile internet access users in that province:


$$\begin{array}{l} Topic\;index_{i}=\frac{Comment\;count_{i}}{Mobile\;internet\;access\;users_{i}} \end{array}$$

This procedure effectively controls for digital penetration bias, allowing for a more equitable comparison of “place-based” health perceptions across regions with varying levels of internet accessibility. Mobile internet access users were selected because they represent the population with the technical capacity to engage with mobile short-video platforms. This denominator does not capture platform-specific user demographics (eg, Douyin’s age and urban-rural composition), which may introduce residual bias.

### Feature Selection via Least Absolute Shrinkage and Selection Operator Regression

On the basis of the theoretical framework established in section 2, we gathered a comprehensive dataset of 8 candidate indicators to represent the demographic, epidemiological, and socioeconomic characteristics of each province. The data were primarily sourced from the National Bureau of Statistics of China and the China Health Statistical Yearbook. To ensure comparability across variables with different units and scales, all independent variables were standardized. The statistical distribution and regional disparities of these standardized candidate variables (*V*_1_*-V*_8_) are visualized in Figure SA-2 in Multimedia Appendix 3. The violin plots reveal significant spatial heterogeneity across the 31 provinces, particularly in economic output (*V*_1_) and HIV epidemiological metrics (*V*_7_ and *V*_8_), providing the necessary empirical variance for spatial analysis.

However, a significant methodological challenge arises from the limited sample size of 31 provincial units. Including all 8 theoretical candidates in the spatial econometric models would severely deplete the degrees of freedom and introduce a substantial risk of overfitting, which could lead to unstable and unreliable parameter estimates. To address this, we used least absolute shrinkage and selection operator (LASSO) regression to perform data-driven feature selection [39]. LASSO was implemented using scikit-learn version 1.8.0 in Python. The regularization parameter λ was selected via 5-fold cross-validation, using λ_min_ (the value minimizing mean squared error). The 1-SE rule (selecting the largest λ within 1 SE of λ_min_) yielded identical selections. By introducing an L1 penalty term, LASSO identifies the most resilient predictors while shrinking the coefficients of redundant or less contributory variables to zero. We conducted multiple iterations of the selection process to evaluate the stability of each variable. As illustrated in Figure SA-3 in Multimedia Appendix 4, four variables demonstrated superior selection frequency.

To ensure the statistical validity of the subsequent modeling, we performed preliminary diagnostics focusing on multicollinearity and spatial dependence. First, a multicollinearity check was conducted for the 4 selected independent variables [40]. As shown in Figure SA-4 in Multimedia Appendix 5, all values remained well below the conservative threshold of 5, indicating that multicollinearity does not pose a threat to the stability of our parameter estimates.

Second, we used GIS-based multipanel mapping to visualize the geographic distribution of these indicators across the 31 provinces. To quantify the observed spatial patterns, we calculated the global Moran *I* index [41]. As labeled in the composite maps (Figure 5), all 4 variables exhibited significant positive spatial autocorrelation. The consistently significant and positive Moran *I* values confirm a high degree of spatial clustering across China’s provincial landscape. These findings provide a definitive statistical justification for transitioning from a nonspatial ordinary least squares (OLS) framework to spatial econometric models (spatial lag model [SLM]/spatial error model [SEM]) to account for spatial dependence and spillover estimated associations.

**Figure 5.**
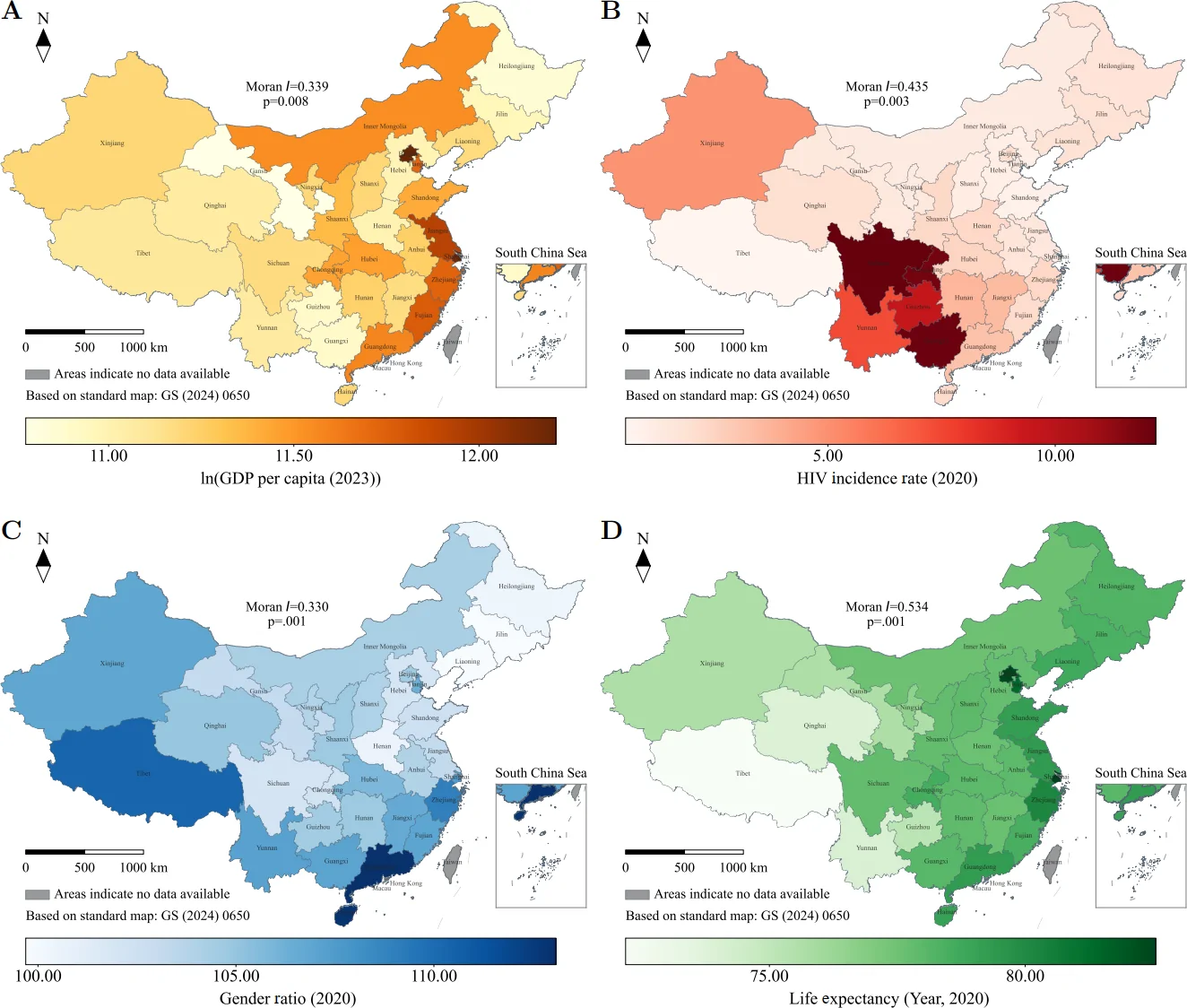
Spatial distribution characteristics of the 4 selected variables across 31 provincial-level units in mainland China. Darker shades indicate higher prevalence, while lighter shades indicate lower prevalence. The global Moran *I* statistic indicates significant spatial clustering.

### Regression Modeling and Robustness

We first estimated an OLS-based multiple linear regression (MLR) model for each thematic outcome to establish a nonspatial benchmark [42]. Model diagnostics were then conducted to evaluate whether this benchmark specification was adequate. Specifically, we applied the Ramsey RESET test to examine potential functional-form misspecification and the Breusch-Pagan test to assess heteroscedasticity [43]. When heteroscedasticity was detected, robust SEs were used to improve the reliability of statistical inference. In addition, we calculated Moran *I* for the OLS residuals to determine whether spatial dependence remained after accounting for the selected socioeconomic and epidemiological predictors.

For topic models exhibiting significant residual spatial autocorrelation, we further implemented spatial econometric models following a general-to-specific strategy [44]. We constructed a queen-contiguity spatial weight matrix W for the 31 provinces using the Queen.from_dataframe method from the libpysal library, in which provinces sharing a common border or vertex were defined as neighbors. The matrix was not row standardized. As a robustness check, we also used an inverse-distance weight matrix ($w_{ij}=1/d_{ij}$) based on provincial centroid distances, and the results were substantively consistent.

The spatial analysis began with the spatial Durbin model (SDM), which accounts for spatial lags in both the dependent variable and the independent variables. We then used Wald and likelihood ratio tests to examine whether the SDM could be simplified into an SLM or an SEM. This procedure allowed us to select a parsimonious spatial specification that captured the relevant form of spatial dependence while avoiding unnecessary overparameterization.

Finally, to control for the inflation of type I errors arising from multiple topic-specific analyses, we applied the false discovery rate (FDR) correction [45]. All *P* values from the final selected models were adjusted using the Benjamini-Hochberg procedure, and associations that remained significant at the FDR-adjusted 0.05 level were reported as robust findings. Given the cross-sectional ecological design of this study, these results should not be interpreted as causal effects. Rather, they characterize regional-level associations between macrocontextual conditions and digital HIV/AIDS discourse and should not be used to infer individual-level psychological mechanisms.

### Interpretation of Localized Contributions via SHAP-GIS

While the MLR and spatial econometric models described earlier provide a robust estimation of “global” average associations across the country, they may mask spatial nonstationarity where the influence of a socioeconomic driver varies significantly from one province to another. To uncover these geographical nuances, we used the SHAP framework to decompose the model’s predictions [46].

Unlike traditional regression coefficients that offer a single aggregate value, the SHAP approach quantifies the marginal contribution of each independent variable for every specific provincial observation [47]. By integrating these province-specific SHAP values with GIS, we generated localized effect maps [48]. This SHAP-GIS coupling allows us to visualize the spatial heterogeneity of the correlates, identifying where and why certain socioeconomic factors (eg, GDP or gender ratio) act as factors associated with increased stigmatizing discourse or inhibitors of specific HIV/AIDS discourses [49]. This transition from global averages to localized interpretation provides the nuanced empirical evidence required for place-based public health interventions. SHAP-GIS was applied only to OLS models that did not exhibit significant spatial autocorrelation (tested via Moran *I* on residuals). For models with significant spatial dependence, we did not apply SHAP-GIS and instead relied on spatial lag and spatial error models for interpretation.

### Ethical Considerations

This study was conducted in accordance with the ethical standards of the responsible committee on human experimentation and with the Declaration of Helsinki. The research protocol, entitled “Exploring Human Behavior in the Social Media Landscape” (principal investigator: PH), was reviewed and approved by the institutional review board of the Hong Kong University of Science and Technology (Guangzhou). The ethics approval reference number was HKUST(GZ)-HSP-2024‐0065. This study exclusively involved the analysis of publicly available social media data. No data were collected directly from human participants, and no identifiable personal information was used or stored during this research. All data handling procedures were performed in strict compliance with the platform’s terms of service and applicable privacy regulations to ensure the confidentiality and anonymity of the data sources.

## Results

### Descriptive Results: The Landscape of Digital Discourse

The thematic classification of the 174,394 collected comments revealed a multifaceted and somewhat polarized public reaction to HIV/AIDS narratives. As summarized in Table 2, the discourse was characterized by a tension between traditional moralizing perspectives and emerging humanitarian support. The most prevalent identifiable theme was moral judgment and attribution of blame (41,663/174,394, 23.89%). This high proportion indicated that HIV/AIDS remains heavily framed through a moral lens in the Chinese digital sphere, with a significant segment of the audience focusing on the “responsibility” or “fault” of the individuals portrayed in the videos.

**Table 2.** Classification results, raw and weighted considered.

Topic name	Count, n (%)
Moral judgment and attribution of blame	41,663 (23.89)
Disease knowledge and transmission discussion	23,344 (13.39)
Support, encouragement, and humanitarian care	18,101 (10.38)
Social impact and public issue discussion	14,908 (8.55)
Humor, banter, and ironic expression	14,580 (8.36)
Risk underestimation and cognitive bias	9326 (5.35)
Worry, fear, and anxiety emotions	7693 (4.41)
Personal experience and current situation sharing	4932 (2.83)
Information sources and credibility discussion	4615 (2.65)
Others	35,232 (20.2)

While disease knowledge and transmission discussion (23,344/174,394, 13.39%) reflected a substantial public desire for medical facts, the presence of risk underestimation and cognitive bias (9326/174,394, 5.35%) suggested that misinformation and a lack of perceived vulnerability persisted among users. Encouragingly, support, encouragement, and humanitarian care (18,101/174,394, 10.38%) emerged as a major pillar of discourse. This suggested that social media also acted as a vital space for prosocial engagement and the expression of empathy toward people living with HIV/AIDS. Emotional expressions ranging from worry and fear (7693/174,394, 4.41%) to humor and banter (14,580/174,394, 8.36%) reflected the complex psychological landscape of the audience. The “Others” category (35,232/174,394, 20.20%) highlighted the vast amount of miscellaneous interaction common on short-form video platforms. A post hoc analysis of 200 randomly sampled comments from this category revealed that the majority comprised platform-specific interactions (83/200, 41.5%), off-topic discussions (56/200, 28.0%), fragmented or ambiguous text (35/200, 17.5%), and comments about video production (26/200, 13%), suggesting that this residual category primarily reflects the diverse interactional nature of short-video discourse rather than undetected stigmatizing content.

### Spatial Distribution of HIV/AIDS Digital Discourse

To uncover the geographic footprint of public health perceptions, we mapped the Topic Index across the 31 provinces. While the full provincial dataset is provided in Figure SA-5 in Multimedia Appendix 6, this section focuses on the spatial patterns of the most prominent themes: moral judgment, support, and disease knowledge. Normalization revealed significant spatial heterogeneity, indicating that a province’s socioeconomic and cultural context deeply influenced the nature of its digital discourse.

As the most prevalent theme, moral judgment exhibited the highest intensity across China; yet, with clear regional disparities. As shown in Figure 6, the highest engagement levels were concentrated in major political and economic centers, notably Beijing (0.4253) and Guangdong (0.3991). These regions acted as provinces with high discursive intensity, where narratives were most likely to be scrutinized through a moral lens. High indices were also observed in Guangxi (0.3370) and Zhejiang (0.3261). Conversely, inland provinces such as Gansu (0.1347) and Qinghai (0.1020) showed significantly lower intensity, suggesting a different cultural or digital framing of HIV narratives in these “places.”

**Figure 6.**
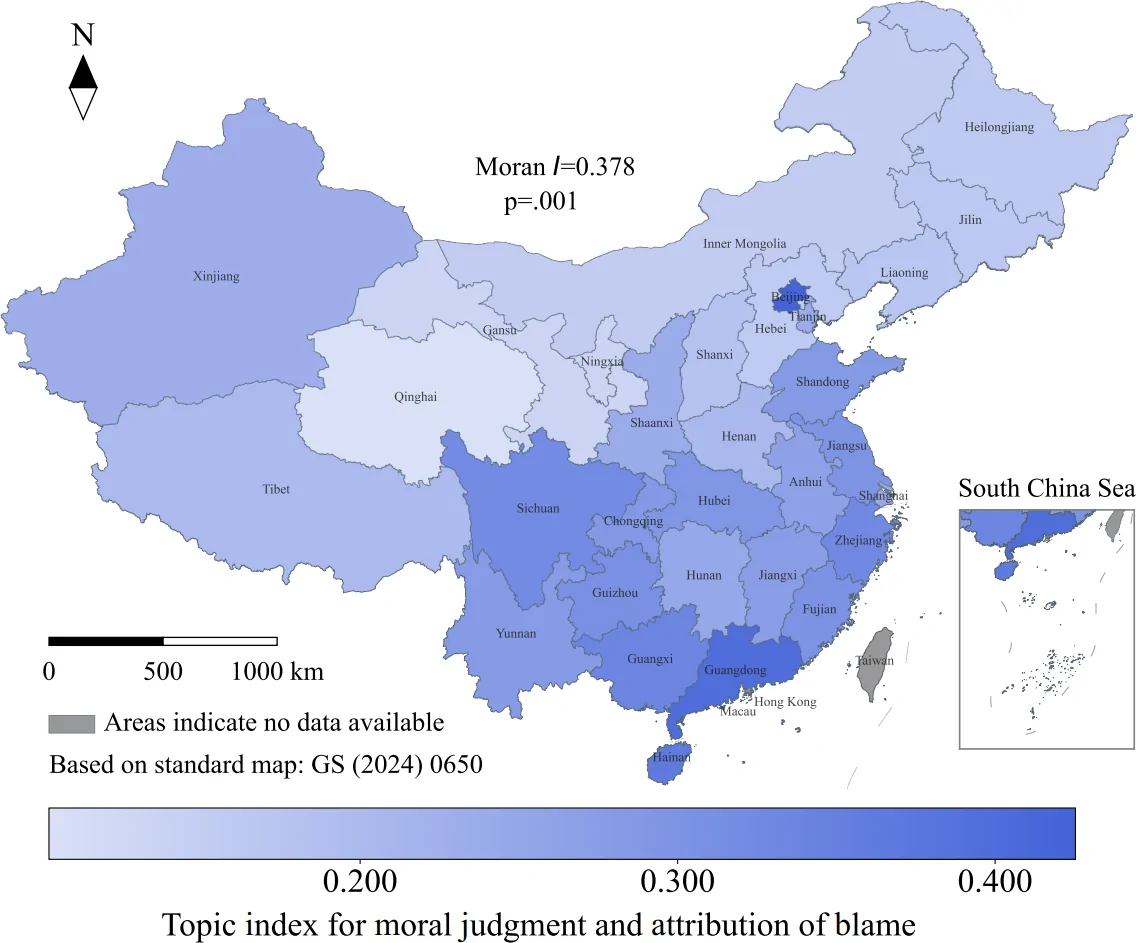
Spatial distribution for the Topic Index of moral judgment and attribution of blame across 31 provincial-level units in mainland China (excluding Taiwan, Hong Kong, and Macao). The index represents the prevalence of moral judgment and attribution of blame comments, calculated as the frequency of comments classified into this theme per 10,000 mobile internet users at the provincial level. Darker shades indicate higher prevalence, while lighter shades indicate lower prevalence. The global Moran *I* statistic indicates significant spatial clustering.

The distribution of supportive social discourse exhibited a distinctive geographical pattern and did not strictly follow economic logic. As shown in Figure 7, Xinjiang stood out with the highest support index (0.2920) in the country. This finding was noteworthy and may reflect a confluence of factors, including region-specific public health messaging, distinct cultural norms regarding health discourse, or platform moderation practices. Further investigation with contextual data is needed to identify the specific mechanisms. Beijing (0.1733) and Yunnan (0.1665) also showed high levels of humanitarian care, reflecting the coexistence of supportive and stigmatizing discourses within the same geographic boundaries. Despite their economic similarities, Jiangsu (0.1102) and Shandong (0.0910) exhibited lower support indices than Guangdong, their southern neighbor.

**Figure 7.**
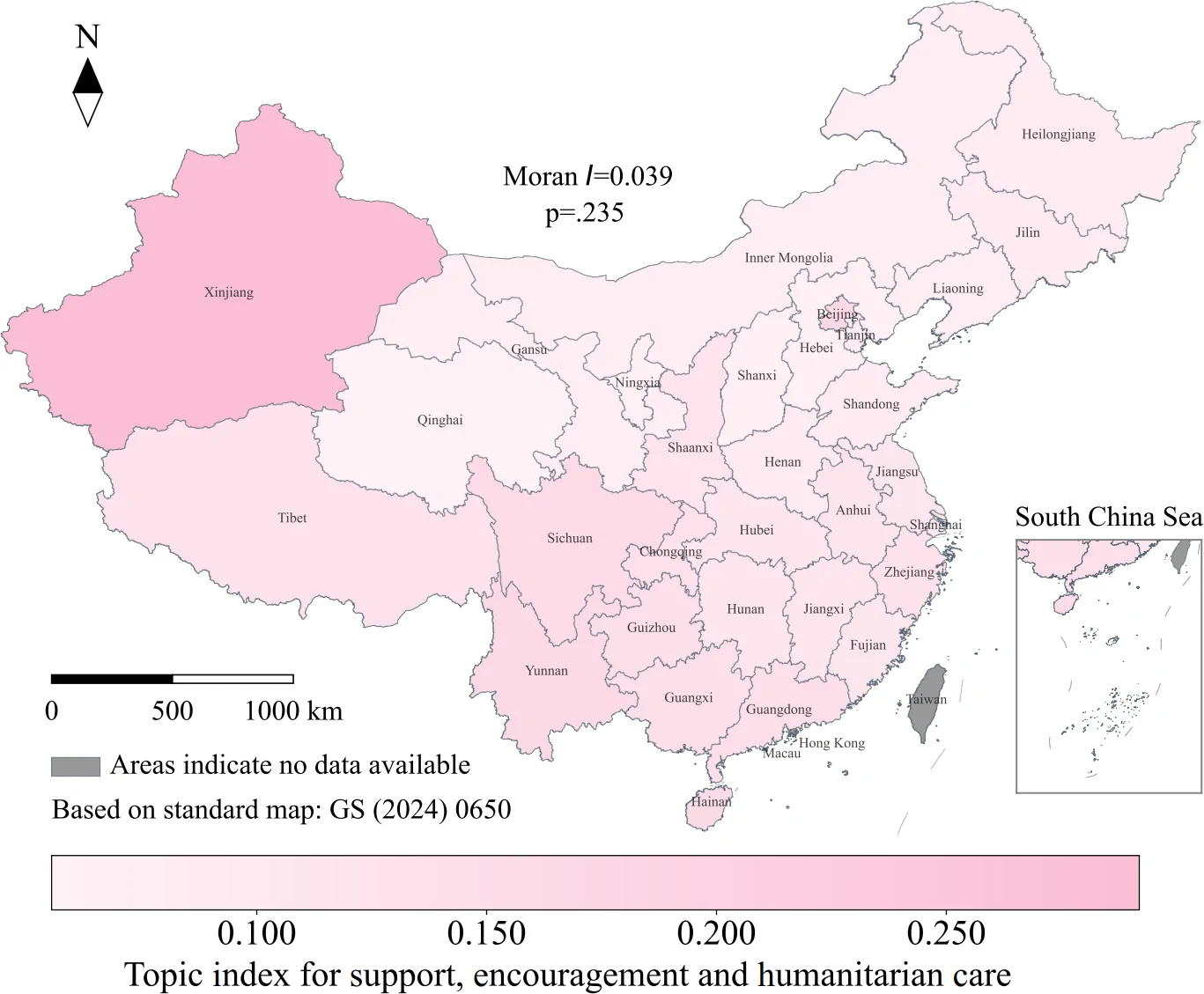
Spatial distribution for Topic Index of support, encouragement, and humanitarian care across 31 provincial-level units in mainland China (excluding Taiwan, Hong Kong, and Macao). The index represents the prevalence of support, encouragement, and humanitarian care comments, calculated as the frequency of comments classified into this theme per 10,000 mobile internet users at the provincial level. Darker shades indicate higher prevalence, while lighter shades indicate lower prevalence. The global Moran *I* statistic does not indicate significant spatial clustering.

Disease knowledge and transmission discussion showed a spatially uneven pattern shaped by both health information capacity and local epidemiological salience. As shown in Figure 8, developed provinces and major digital hubs, including Guangdong (0.2294), Beijing (0.2137), Zhejiang (0.1856), and Jiangsu (0.1717), exhibited relatively high Topic Indices, possibly reflecting stronger health literacy, information access, and digital engagement. Meanwhile, several provinces with higher HIV epidemiological salience, such as Guangxi (0.2075), Sichuan (0.1960), Chongqing (0.1923), Yunnan (0.1578), and Xinjiang (0.1293), also showed elevated levels of knowledge-oriented discussion, suggesting that public information demand may be stronger where HIV/AIDS is more visible. In contrast, Qinghai (0.0554) and Gansu (0.0654) reported the lowest intensities, indicating weaker normalized engagement with medical and transmission-related HIV/AIDS information.

**Figure 8.**
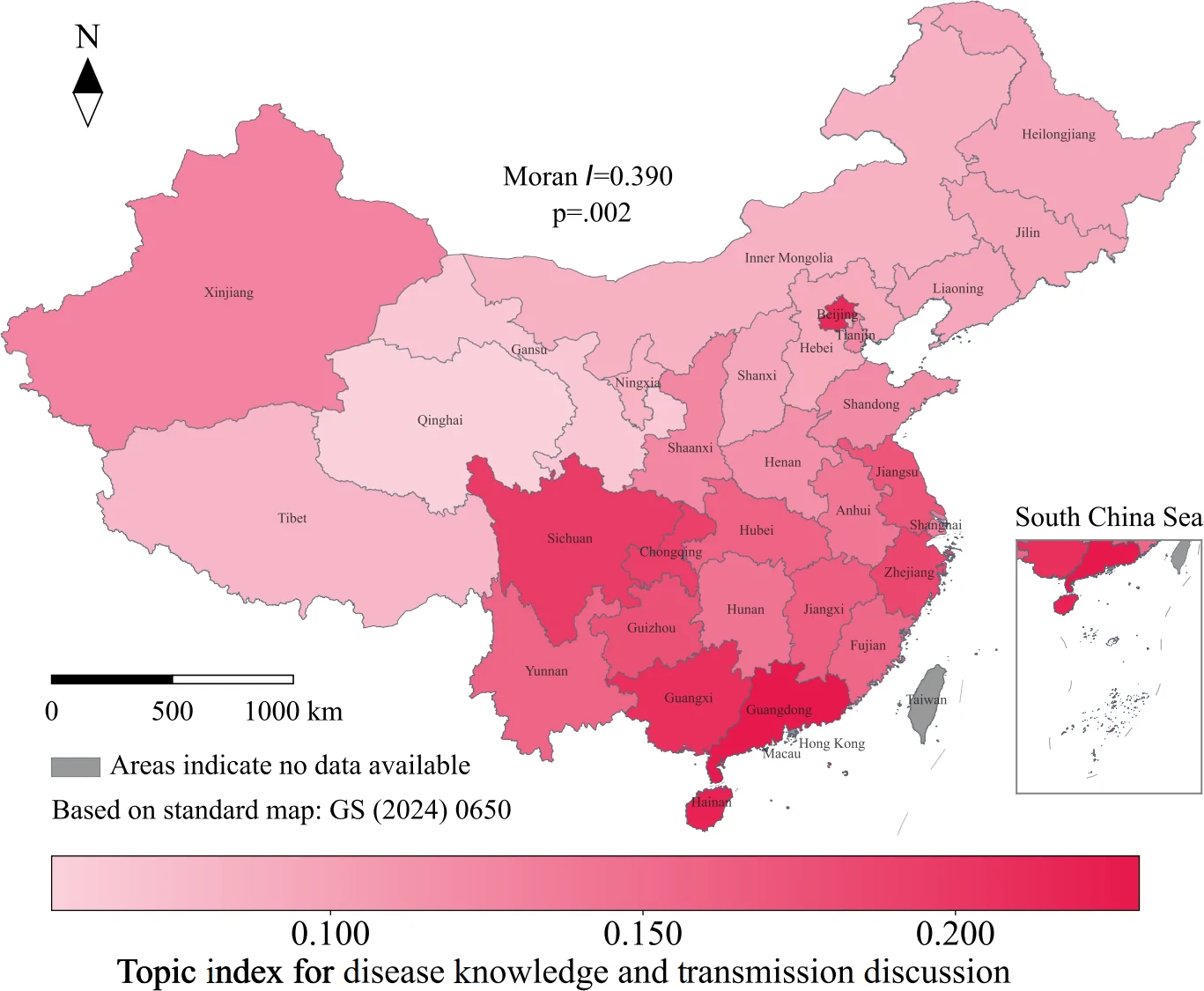
Spatial distribution of disease knowledge and transmission discussion across 31 provincial-level units in mainland China (excluding Taiwan, Hong Kong, and Macao). The index represents the prevalence of disease knowledge and transmission discussion comments, calculated as the frequency of comments classified into this theme per 10,000 mobile internet users at the provincial level. Darker shades indicate higher prevalence, while lighter shades indicate lower prevalence. The global Moran *I* statistic indicates significant spatial clustering.

### Regression Analysis: Sociohealth Correlates of Digital Discourse

#### Overview

To systematically identify the correlates of provincial discourse, we estimated regression models for 9 thematic topics. The core results, integrated with spatial diagnostics and multiple-testing corrections, are synthesized in the FDR-corrected standardized β heatmap (Figure 9). Owing to the large volume of regression outputs (9 topic models×multiple predictors), full results, including all coefficients, SEs, CIs, *P* values, and model fit statistics, are provided on GitHub [35]. The models achieved substantial explanatory power, with *R*^2^ (McFadden *R*^2^ for the SLM) values ranging from 0.403 (support) to 0.842 (personal experience). Diagnostic tests identified significant spatial autocorrelation in the residuals for personal experience, risk underestimation, and worry and anxiety. Consequently, an SLM was used for these topics (labeled as [Lag] in Figure 9) to account for spatial dependence and potential spillover patterns among neighboring provinces. By applying the FDR correction, we ensured that the reported associations were robust against type I errors. Furthermore, for topics such as moral judgment where heteroscedasticity was detected ([H]), robust SEs were used to ensure the validity of our inferences.

**Figure 9.**
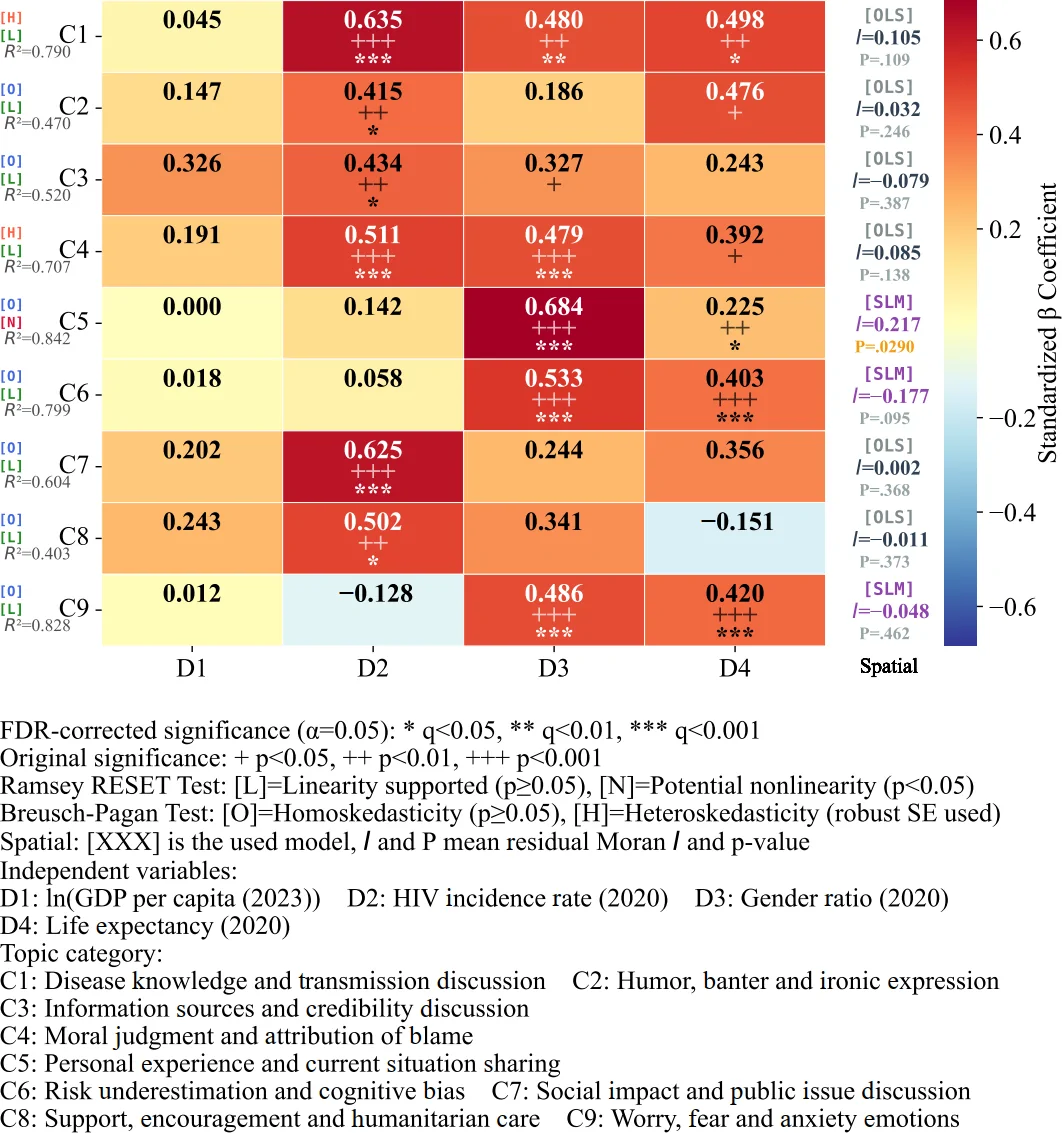
Predictive effects of socioeconomic and health indicators (D1-D4) on HIV/AIDS–related social media discussion topics (C1-C9). The matrix displays standardized regression coefficients with false discovery rate (FDR)–corrected significance levels (*, **, ***), heteroscedasticity tests ([O]/[H]), and linearity tests ([L]/[N]). The spatial analysis column reports residual Moran *I* values and the final spatial regression approach (eg, SLM). GDP: gross domestic product; OLS: ordinary least squares; SLM: spatial lag model.

The HIV incidence rate emerged as the most consistent and powerful driver across multiple discourse dimensions, reinforcing the “contact theory” in a digital context. Higher incidence rates were most strongly associated with disease knowledge (β=.635; *q*<.001) and social impact discussion (β=.625; *q*<.001). Crucially, it was the primary factor associated with increased stigmatizing discourse for moral judgment (β=.511; *q*<.001), suggesting that in provinces with higher disease visibility, the public was more likely to engage in both factual inquiry and moralistic scrutiny. The HIV incidence rate was also positively associated with support, encouragement, and humanitarian care (β=.502; *q*=.012), indicating that higher epidemiological salience corresponded not only to stronger stigmatizing discourse but also to greater prosocial engagement. This pattern suggested that disease visibility may intensify public attention in multiple directions, producing both moralized scrutiny and humanitarian responses.

The gender ratio representing the demographic substrate of a province showed a unique influence on subjective themes. Provinces with higher male-to-female ratios were significantly more likely to engage with personal experience sharing (β=.684; *q*<.001) and risk underestimation (β=.533; *q*<.001). This suggested that the demographic composition of a “place” may shape the communal appetite for anecdotal versus factual content. This also contributed significantly to moral judgment (β=.479; *q*<.001), hinting that demographic imbalances may correlate with more rigid or judgment-heavy social norms in digital spaces.

As a proxy for public health resilience, higher life expectancy was positively associated with disease knowledge (β=.498*; q*=.018) and humor and banter (β=.476; *P*=.03). This suggested that in regions with superior health capital, the public might feel more “empowered” to discuss HIV through either intellectual inquiry or social levity. Surprisingly, ln(GDP per capita) showed relatively modest standardized β values compared to epidemiological and demographic factors. Its most notable (although marginally significant) association was on support (β=.243; *P*=.32), suggesting that economic prosperity provided a baseline for humanitarian expression but did not dictate the emotional tone of the discourse as strongly as the actual disease burden did.

#### Localized Correlates of Discourse: SHAP-GIS Interpretation

While the previous section established global associations, the global coefficients might mask significant spatial heterogeneity. We applied the SHAP-GIS framework to the OLS models of 3 representative topics to visualize the “local force” exerted by key socioeconomic correlates across different provinces.

#### Localized Association of HIV Incidence on Moral Judgment

The SHAP-GIS analysis for moral judgment and attribution of blame (Figure 10) revealed a clear geographical divide in the localized association between HIV incidence rate and moralizing discourse. While the global standardized β coefficient indicated a strong positive association between HIV incidence and moral judgment (β=.511; *q*<.001), the localized SHAP values, ranging from −0.432 to 1.336, showed that this association varied substantially across provinces. The highest positive SHAP values were concentrated in Southwest China. Sichuan (SHAP=1.336), Guangxi (SHAP=1.313), Chongqing (SHAP=1.287), and Guizhou (SHAP=0.940) exhibited the largest positive localized contributions of HIV incidence rate to the predicted intensity of moral judgment and attribution of blame. In these provinces, relatively high HIV incidence values, such as Sichuan at 12.21 per 100,000 population, were associated with stronger model-predicted moralizing discourse. This pattern suggested that in high-prevalence provinces, epidemiological salience might coincide with stronger moral attribution in digital HIV/AIDS discussions.

**Figure 10.**
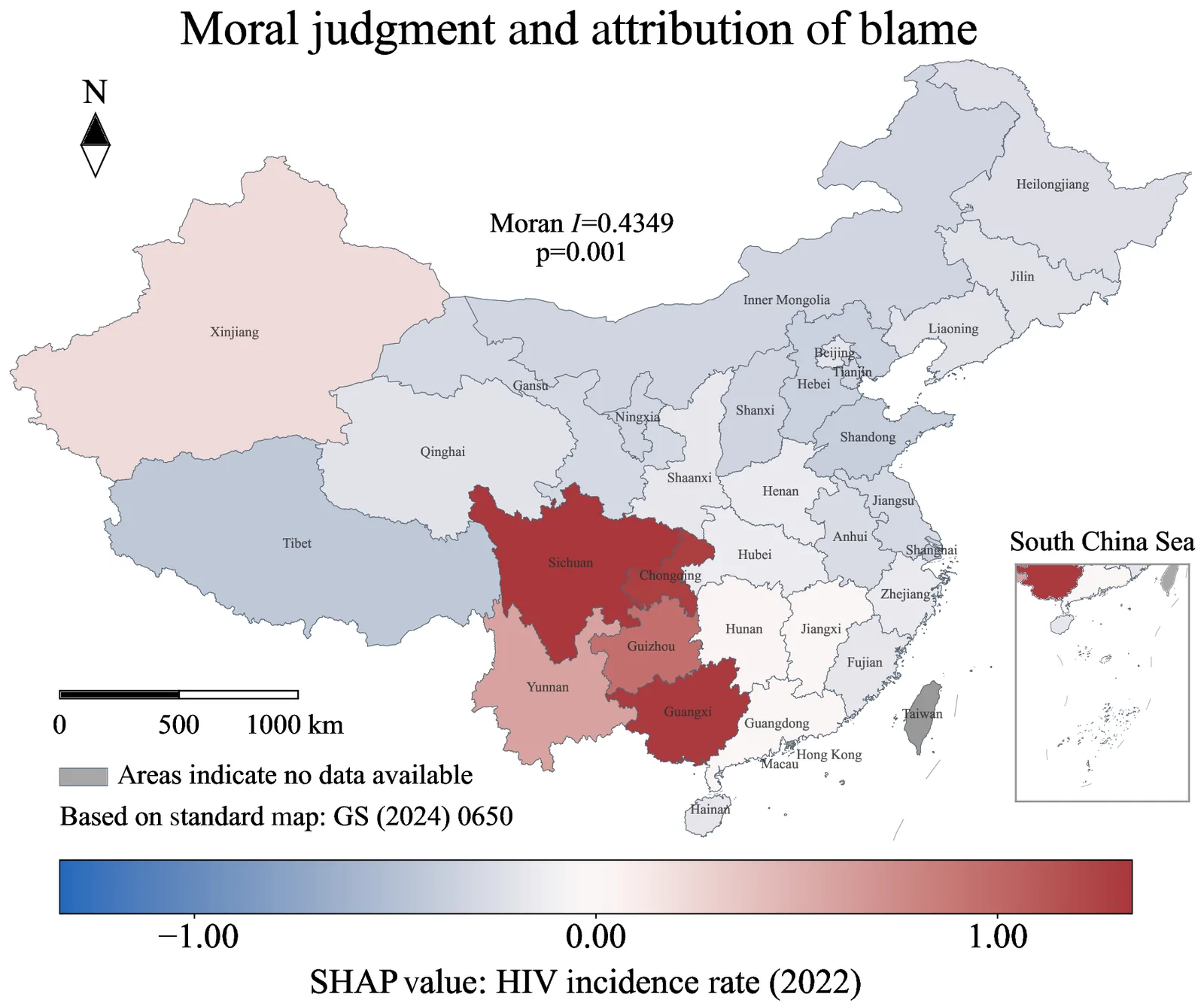
Spatial distribution of localized Shapley Additive Explanations (SHAP) values for HIV incidence rate on moral judgment and attribution of blame. The color gradient represents the magnitude of the SHAP values (blue=buffering and red=amplifying), and the global Moran *I* statistic indicates significant spatial clustering.

Conversely, in regions with lower epidemiological visibility, HIV incidence rate showed negative localized SHAP values for moral judgment. Tibet (−0.432), Shandong (−0.384), and Shanghai (−0.346) exhibited the most negative SHAP values. In these contexts, relatively low HIV incidence values, such as 0.55 per 100,000 population in Tibet, were associated with lower model-predicted levels of moralizing discourse. This pattern indicated that lower local epidemiological salience corresponded to weaker moral attribution in the model. Notably, Yunnan, despite its historically high disease burden (7.37 per 100,000 population), showed a more moderate SHAP value (0.602) compared with neighboring provinces such as Sichuan and Guangxi. This relative moderation suggested that the relationship between HIV incidence and moral judgment might be shaped by additional contextual factors, such as long-term public health communication, local awareness, or region-specific social responses to HIV/AIDS. However, these possible explanations should be interpreted cautiously, as the present cross-sectional ecological design cannot establish causal mechanisms.

#### Localized Association of Demographic Substrate on Moral Judgment

As identified in the LASSO selection process, gender ratio was one of the most stable demographic predictors of digital HIV/AIDS discourse. Figure 11 shows that the localized contribution of gender ratio exhibited a marked regional divide, with SHAP values ranging from −0.770 to 1.174. Provinces with relatively male-skewed demographic structures tended to show positive localized SHAP values for moral judgment and attribution of blame. Guangdong (SHAP=1.174; feature=113.08) and Hainan (SHAP=1.142; feature=112.86) exhibited the strongest positive localized contributions. Positive SHAP values were also observed in Tibet (0.773) and Zhejiang (0.587). In these provinces, higher male-to-female ratios were associated with higher model-predicted intensity of moral judgment.

**Figure 11.**
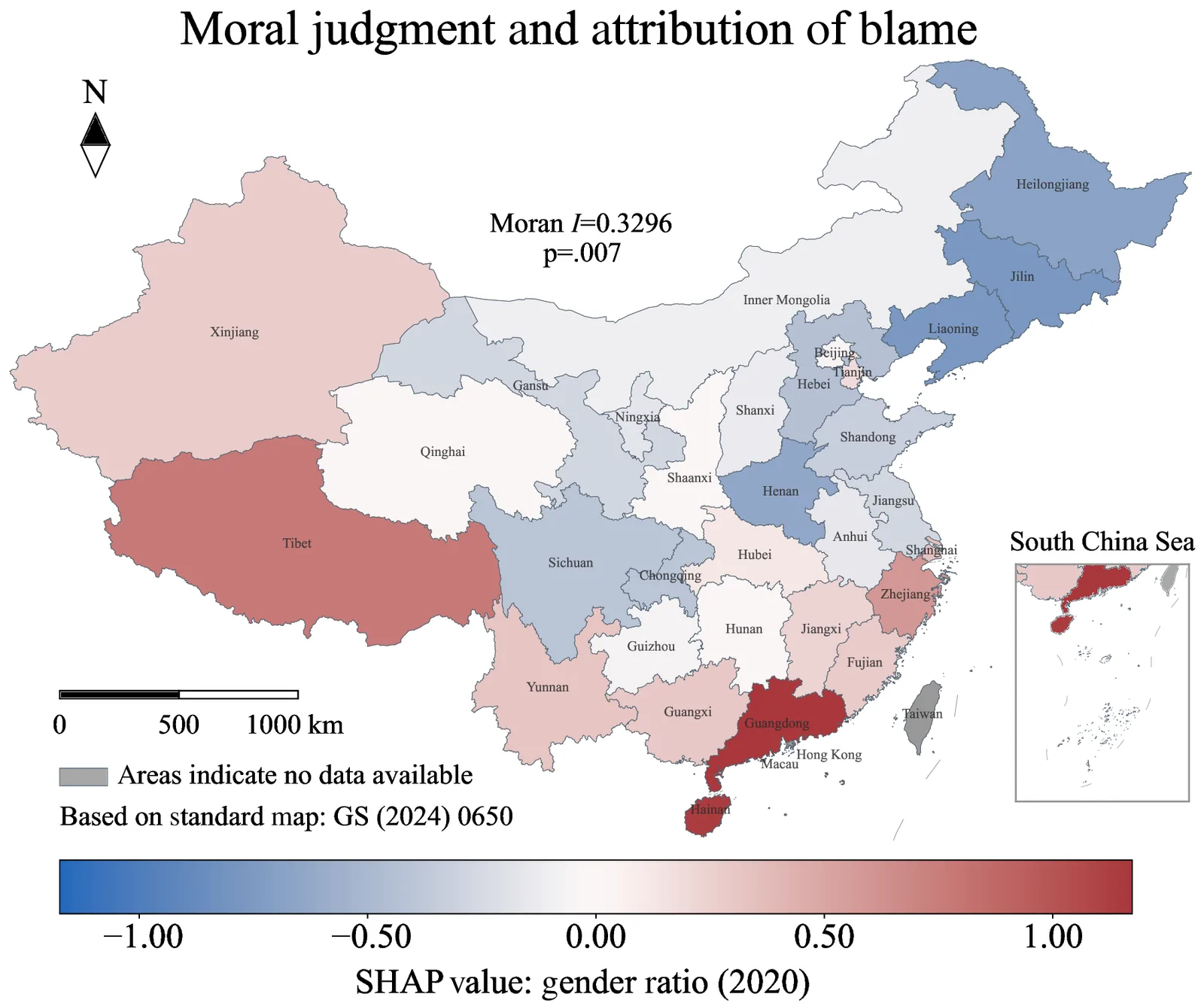
Spatial distribution of localized Shapley Additive Explanations (SHAP) values for gender ratio on moral judgment. The color gradient represents the magnitude of the SHAP values (blue=buffering and red=amplifying), and the global Moran *I* statistic indicates significant spatial clustering.

Conversely, negative localized SHAP values were observed in Northern China, particularly in the Northeast. Jilin (−0.770), Liaoning (−0.769), and Heilongjiang (−0.675) exhibited the most negative SHAP values in the country. These provinces also had relatively low gender ratios, such as 99.69 in Jilin, indicating a slightly female-skewed or more balanced population structure. In these contexts, a more balanced demographic composition was associated with lower model-predicted moral judgment. These patterns suggested that demographic structure was spatially heterogeneous in its association with digital HIV/AIDS stigma, but further research is needed to clarify the social mechanisms behind this relationship.

## Discussion

### Principal Findings

This study revealed that discussions on Chinese social media platforms about the proactive sharing of daily-life narratives by people living with HIV/AIDS exhibited a complex and multifaceted structure across different regions and socioeconomic contexts. The following interpretations, while grounded in our empirical findings, were offered as hypotheses for future research and should not be considered definitive explanations.

### Thematic Landscape of Digital HIV/AIDS Discourse: From Moral Judgment to Supportive Care

The thematic classification of more than 170,000 comments revealed that the Chinese digital sphere remained a contested terrain for HIV/AIDS discourse. The dominance of moral judgment (23.89%) suggested that traditional stigmatization and “othering” processes remain deeply rooted in public perception. However, the substantial presence of humanitarian support (10.38%) and disease knowledge (13.39%) indicated a growing counter-discourse characterized by health literacy and digital empathy. This pluralistic landscape reflected a transition in public health communication, where short-form video platforms such as Douyin provide a space in which old stigmas and empathy-oriented public discourse coexist [50].

### Spatial Heterogeneity of Stigma- and Support-Oriented Discourse

Our GIS analysis indicated that “place” was associated with the intensity of these digital interactions. The high Topic Index for moral judgment in major hubs such as Beijing and Guangdong suggested that high digital penetration did not inherently correspond to more progressive discourse; rather, it might coincide with stronger expressions of existing social tensions [51]. The high engagement with both knowledge and stigma in provinces such as Guangxi and Sichuan is consistent with the possible relevance of localized disease visibility [16]. The elevated level of support in Xinjiang deviates from national economic trends, suggesting that specific regional cultural narratives, public health campaigns, or viral events may be associated with distinctive discourse environments within the digital landscape. Further research with contextual data is needed to identify the underlying mechanisms.

### Sociohealth Correlates in the Geography of Digital HIV/AIDS Stigma

The global regression analysis highlighted the associated role of HIV incidence and gender ratio in relation to digital discourse. Higher incidence was associated with both knowledge seeking and increased moral judgment [52]. This was consistent with the hypothesis that proximity to the epidemic might be associated with a defensive “risk-distancing” response, where moral attribution may appear alongside heightened disease visibility [53]. We noted that our operationalization of epidemiological proximity, provincial HIV incidence rate, differs from traditional intergroup contact measured at the individual level. We interpreted these ecological associations as evidence that epidemiological salience might be related to discursive environments through visibility and perceived risk, rather than as a direct test of interpersonal contact theory [26]. The selection of gender ratio as the top LASSO predictor reflected the association between population structure and social norms [54]. In male-skewed demographic “places,” the discourse tended to be more rigid and judgment heavy, identifying gender balance as a crucial, although often overlooked, indicator of a region’s discourse environment.

### Gender Balance and Moralized Health Norms

Our global and localized analyses underscored gender ratio as a consistently strong correlate of HIV/AIDS stigma. The robust SHAP-GIS findings regarding the north-south divide suggested that “place” in digital China is partly associated with its demographic substrate. The higher level of moral judgment in the male-skewed South versus the relatively lower moral judgment intensity in the balanced Northeast indicated that gendered social environments were associated with the “cultural baseline” for empathy and blame. However, we noted that in provinces such as Guangdong, the elevated gender ratio was substantially driven by large floating or migrant populations rather than birth-cohort imbalances alone. The intersection of demographic skew with population mobility and urbanization might be jointly associated with discourse environments. Future studies should disentangle the relative contributions of demographic structure and migration patterns. This suggested gender balance not merely as a demographic statistic, but as a potentially important social factor in health communication that warrants further investigation [19].

### Policy Implications for Place-Based Digital Health Governance

Before discussing specific implications, it is important to clarify what we mean by “stigma” in the context of intervention. In this study, we operatically implement stigma as expressions of social disapproval, blame attribution, and negative stereotyping toward people living with HIV/AIDS in publicly observable online discourse. This reflects both the presence of objective misconceptions among the commenting public and the discursive environment that people living with HIV/AIDS may perceive as stigmatizing. Our data do not distinguish between patients’ perceived stigma and nonpatients’ expressed prejudice, but we treat the 2 as interrelated: expressed stigma in public discourse may contribute to the perceptual environment that shapes patients’ lived experiences.

Our findings identify a “stigma-visibility” association in Southwest China, where elevated HIV incidence is correlated with heightened moral judgment. This pattern may be interpreted through the lens of defensive attribution theory: in high-prevalence provinces such as Sichuan and Guangxi, the epidemiological context may be associated with a psychological distancing pattern [55]. By framing HIV/AIDS through a lens of individual moral culpability, the digital public may symbolically distance the disease from the self, thereby reinforcing an “othering” frame around the disease [56]. As the public in these regions may use moral attribution as a form of perceived-risk distancing [52], digital campaigns should prioritize humanizing the narratives of people living with HIV/AIDS to reduce the “othering” process associated with defensive stigma [57].

The high discursive intensity in tier-1 cities such as Beijing and Shanghai creates a unique challenge: these hubs show both high empathy and high moral scrutiny. Platforms must move beyond simple content moderation. In these polarized “intensity hubs,” algorithms should be calibrated to promote constructive advocacy over moralistic conflict, helping ensure that high digital engagement is associated with social inclusion rather than discursive noise [15]. Policymakers should leverage the high digital literacy in these regions to pilot innovative “buddy programs” and volunteer networks that can bridge traditional social gaps.

The robust association between gender ratio and moral judgment, with positive SHAP contributions clustered in the male-skewed South and West, highlights the relevance of social structure in health communication [58]. In provinces such as Guangdong and Hainan, antistigma campaigns must account for gendered social norms. Messaging should explicitly challenge rigid moral frameworks often observed in male-skewed demographic environments. Encouraging a more diverse range of voices in digital discourse may help buffer against moralized reactions associated with demographic imbalances [59].

### Limitations and Future Work

While this study offered a novel spatial analysis of digital HIV/AIDS discourse, several limitations warrant acknowledgment. First, our analyses were conducted at the provincial level (N=31), and the observed associations between provincial characteristics and discourse patterns should not be interpreted as individual-level psychological processes due to the risk of ecological fallacy. Multilevel studies with individual-level data are needed to validate these ecological patterns.

Second, social media data were subject to self-selection bias, as digital discourse is predominantly shaped by a “vocal minority,” potentially excluding the perspectives of the “silent majority.” The keyword “抗艾日常” specifically attracted users interested in or affected by HIV/AIDS, and our findings reflected the discourse patterns of Douyin users engaging with people living with HIV/AIDS daily-life narratives rather than representing the broader Chinese digital public. Additionally, provincial engagement with HIV-related content might not be uniform, as variation in local public health campaigns, disease prevalence, and awareness initiatives might influence comment volume and thematic composition independently of actual public attitudes.

Third, despite using advanced natural language processing techniques, automated text analysis has inherent limitations in capturing linguistic nuances such as sarcasm, irony, and localized dialects. The 6-character filtering threshold may have systematically excluded shorter supportive expressions, such as “加油.” Fourth, reliance on a single platform, Douyin, introduces data constraints, as its user demographics and algorithmic dynamics may differ from other platforms. IP-based geolocation might not reflect users’ region of primary socialization, particularly for migrant workers, introducing measurement errors in province-level discourse assignment.

Fifth, the SHAP-GIS framework provided a cross-sectional view but did not capture temporal dynamics of public health perceptions. Sixth, the normalization of Topic Index using mobile internet access users might introduce systematic bias if platform-specific user demographics, such as age and urbanicity, vary systematically across provinces. Seventh, while LASSO mitigates overfitting, the small sample size (N=31) remained a constraint for the stability of parameter estimates, and our SHAP-GIS localized estimated contributions should be interpreted as exploratory rather than confirmatory.

Several avenues for future inquiry emerge from these limitations. Researchers could integrate digital analysis with offline household surveys to cross-validate findings against more representative population samples. The adoption of multimodal models capable of simultaneously analyzing video content and text would better capture emotional subtexts in short-form video interactions. Multiplatform comparative studies encompassing Weibo, Xiaohongshu, and other social media would offer a more comprehensive view of the national digital health landscape. Finally, incorporating longitudinal data would enable tracking how the “geography of stigma” evolves over time, providing dynamic evidence for place-based public health interventions.

### Conclusions

By integrating LLM-based text classification, LASSO-regularized regression, spatial econometrics, and SHAP-GIS decomposition, this study offered a multidimensional perspective on how HIV/AIDS narratives were consumed and judged across China. This study leveraged LLMs to enable large-scale annotation of social media content related to HIV discourse, enhancing the efficiency of text classification in computational social science research. Methodologically, we integrated “big data” classification with “small sample” provincial analysis, providing a framework for addressing spatial nonstationarity in social media health data. Theoretically, our findings suggested that the associations between sociodemographic and epidemiological factors and digital discourse are geographically contingent rather than universally uniform. Empirically, we identified specific provincial patterns, revealing a complex digital landscape characterized by elevated stigma discourse in southwestern provinces, higher supportive discourse in certain regions, and a north-south demographic association with moral judgment.

## Supplementary material

10.2196/93007 Multimedia Appendix 1 Distinguishing stigmatizing from nonstigmatizing content.

10.2196/93007 Multimedia Appendix 2 Confusion matrix for large language model classification validation.

10.2196/93007 Multimedia Appendix 3 Data distribution of candidate variables.

10.2196/93007 Multimedia Appendix 4 Ranking of variable selection frequency in least absolute shrinkage and selection opLeast Absolute Shrinkage and Selection Operator regression.

10.2196/93007 Multimedia Appendix 5 Variance inflation factor test results for the 4 selected variables.

10.2196/93007 Multimedia Appendix 6 Topic Index spatial distribution heat map.

## Abbreviations

FDR: false discovery rate
GDP: gross domestic product
GIS: geographic information system
LASSO: least absolute shrinkage and selection operator
LLM: large language model
MLR: multiple linear regression
OLS: ordinary least squares
RQ: research question
SDM: spatial Durbin model
SEM: spatial error model
SHAP: Shapley Additive Explanations
SLM: spatial lag model
UNAIDS: Joint United Nations Programme on HIV/AIDS
