# Supplementary material for: Spatial Heterogeneity of Digital HIV/AIDS Stigma in China: Cross-Sectional Ecological Study
Source: J Med Internet Res. 2026 Oct 9;28:e93007. doi: 10.2196/93007 (PMC13652526; doi:10.2196/93007)
Supplement: Supplementary file 1 [file jmir-v28-e93007-s001.pdf]

Multimedia Appendix 6 Distinguishing Stigmatizing from Non-Stigmatizing Content

This appendix supplements the thematic category definitions and representative examples provided in the Text Classification section of the main text. It clarifies the distinction between stigmatizing content and negative-in-tone but factually accurate content.

In this study, a comment was classified as stigmatizing only when it met at least one of the following criteria: **Explicit blame attribution**: attributing moral fault or personal responsibility to PLWHA for their infection or condition; **Derogatory or dehumanizing language**: using insulting, degrading, or dehumanizing terms toward PLWHA as individuals or as a group; **Social exclusion or distancing**: expressing rejection, avoidance, or calls for segregation of PLWHA from community or social life.

The following examples illustrate comments that met the above criteria:

- Except for those infected by blood transfusion, others do not deserve sympathy.
- People like you shouldn't be near children.
- AIDS is a death sentence; why are you still here?

By contrast, the following comments were not classified as stigmatizing:

- Stay strong, we support you.
- With proper treatment, HIV is now a chronic condition.
- You are brave for sharing your story.

Importantly, comments that conveyed negative health information without moral judgment were not classified as stigmatizing. For example, a comment stating "HIV/AIDS currently has no cure" states a factual limitation but does not attribute blame or express social exclusion toward

PLWHA. Similarly, comments discussing transmission risks or prevention measures without derogatory language were classified into the "Disease Knowledge" category rather than as stigma. The validation sample (95.20*% accuracy, Cohen's* $\kappa = 0.9467$*) confirmed that the model reliably applied this distinction.*

For completeness, we also provide examples of non-stigmatizing content from other thematic categories:

Disease Knowledge: "With proper treatment, HIV is now a chronic condition; no need to panic."

Personal Experience: "I have been on medication for years and live a normal life."

Support and Encouragement: "You are not alone; we are all with you."

Full category definitions and additional examples are available in the Text Classification section of the main text.
