# Supplementary material for: Spatial Heterogeneity of Digital HIV/AIDS Stigma in China: Cross-Sectional Ecological Study
Source: J Med Internet Res. 2026 Oct 9;28:e93007. doi: 10.2196/93007 (PMC13652526; doi:10.2196/93007)
Supplement: Supplementary file 2 [file jmir-v28-e93007-s002.pdf]

Multimedia Appendix 1 Confusion Matrix for LLM Classification Validation

The model achieved 95.20% overall accuracy with Cohen's $\kappa = 0.9467$. Rows represent true labels (human-coded); columns represent predicted labels (DeepSeek R1 671B).

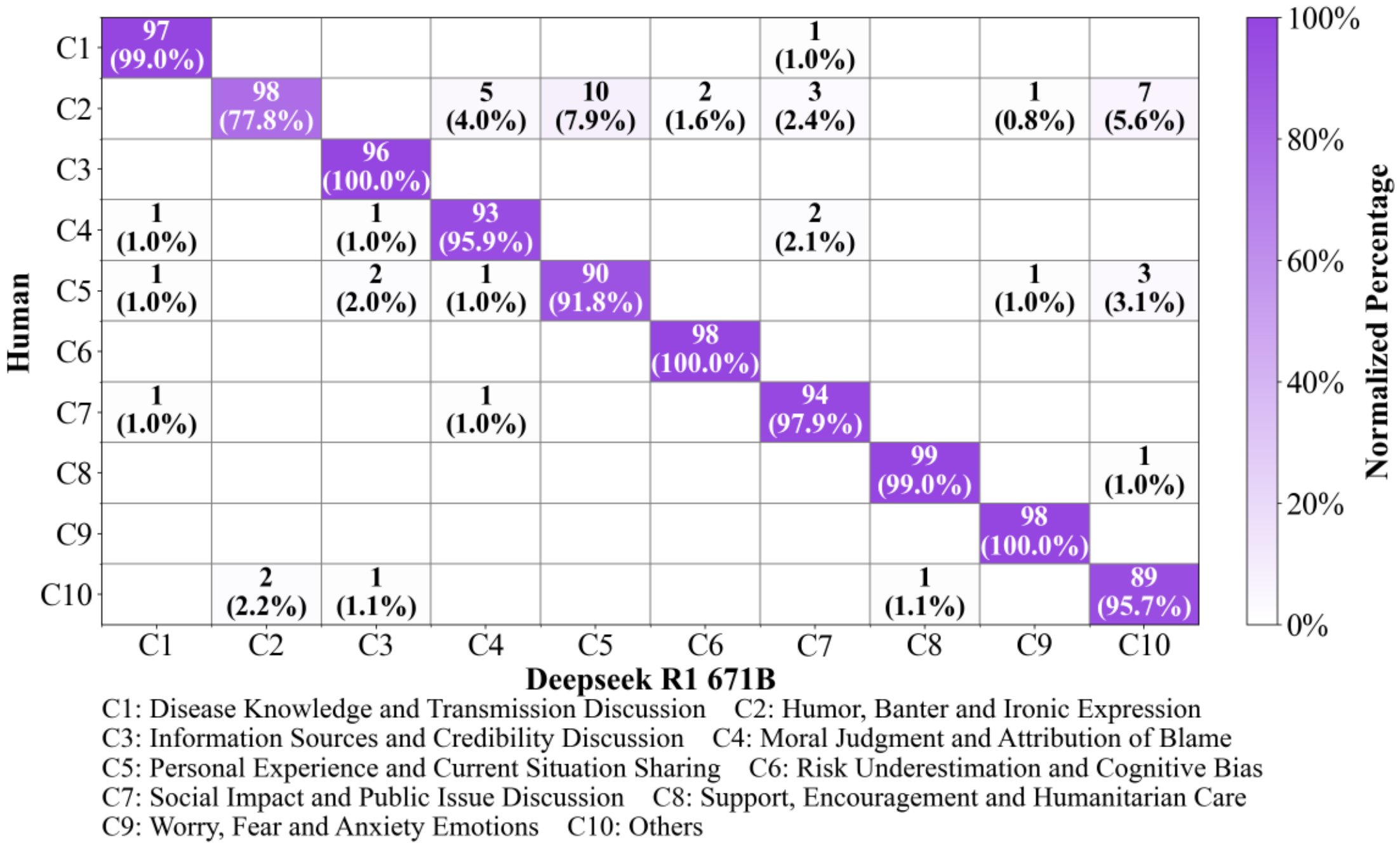

Figure A.1 Confusion matrix for the stratified validation sample (N = 1,000; 100 per category)
