# Supplementary material for: Spatial Heterogeneity of Digital HIV/AIDS Stigma in China: Cross-Sectional Ecological Study
Source: J Med Internet Res. 2026 Oct 9;28:e93007. doi: 10.2196/93007 (PMC13652526; doi:10.2196/93007)
Supplement: Supplementary file 3 [file jmir-v28-e93007-s003.pdf]

Multimedia Appendix 2 Data Distribution of Candidate Variables

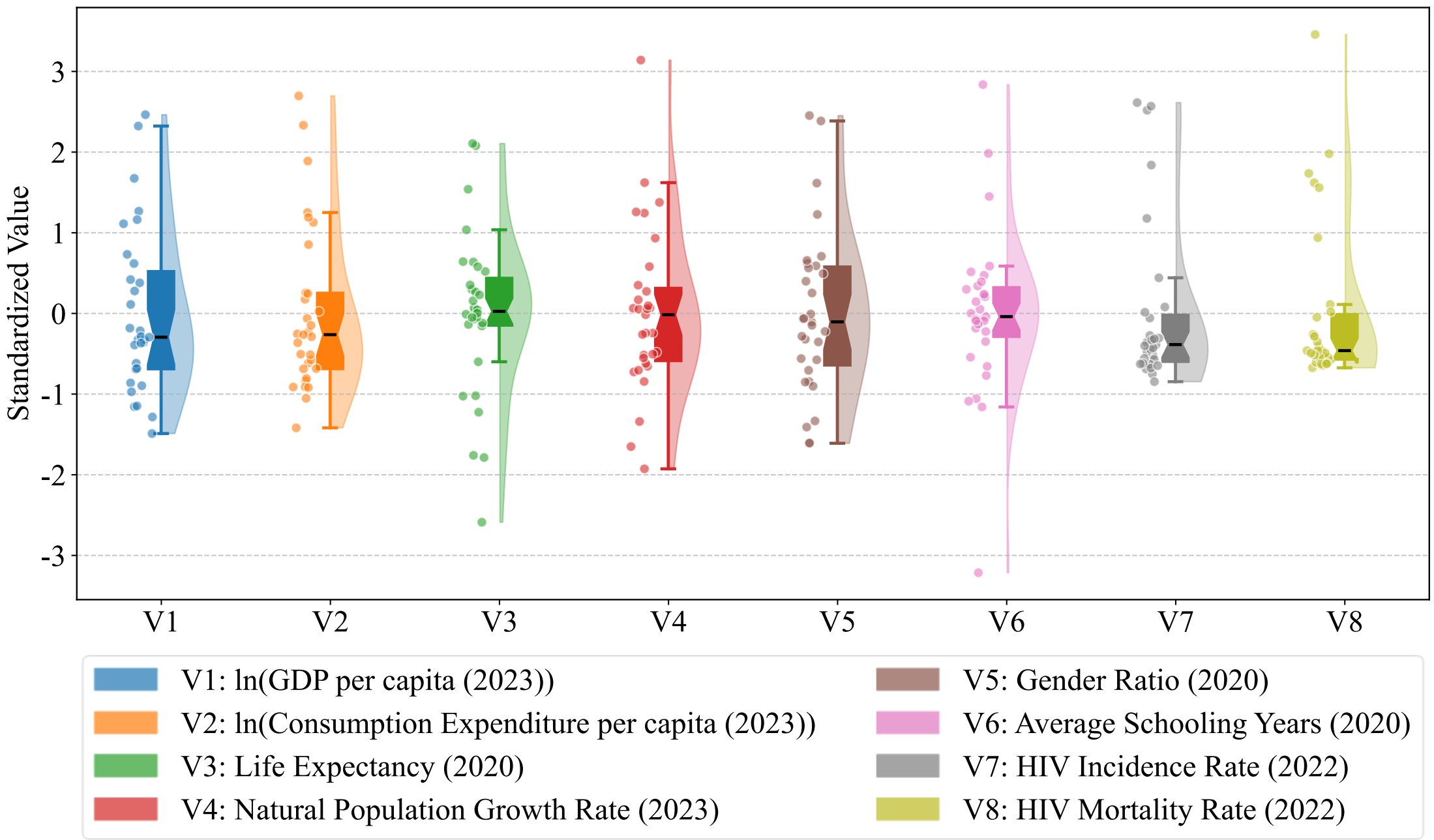

Figure A.2 Data Distribution of Eight Candidate Variables
