# Supplementary material for: Spatial Heterogeneity of Digital HIV/AIDS Stigma in China: Cross-Sectional Ecological Study
Source: J Med Internet Res. 2026 Oct 9;28:e93007. doi: 10.2196/93007 (PMC13652526; doi:10.2196/93007)
Supplement: Supplementary file 4 [file jmir-v28-e93007-s004.pdf]

Multimedia Appendix 3 Ranking of Variable Selection Frequency in LASSO Regression

We performed 5-fold cross-validated LASSO regression for each of the nine topic models. Variables with non-zero coefficients across all five folds were retained. The complete LASSO results, including selection frequency and coefficients for each variable across all nine topics, are provided in Github (https://github.com/cauzp/JMIR90037).

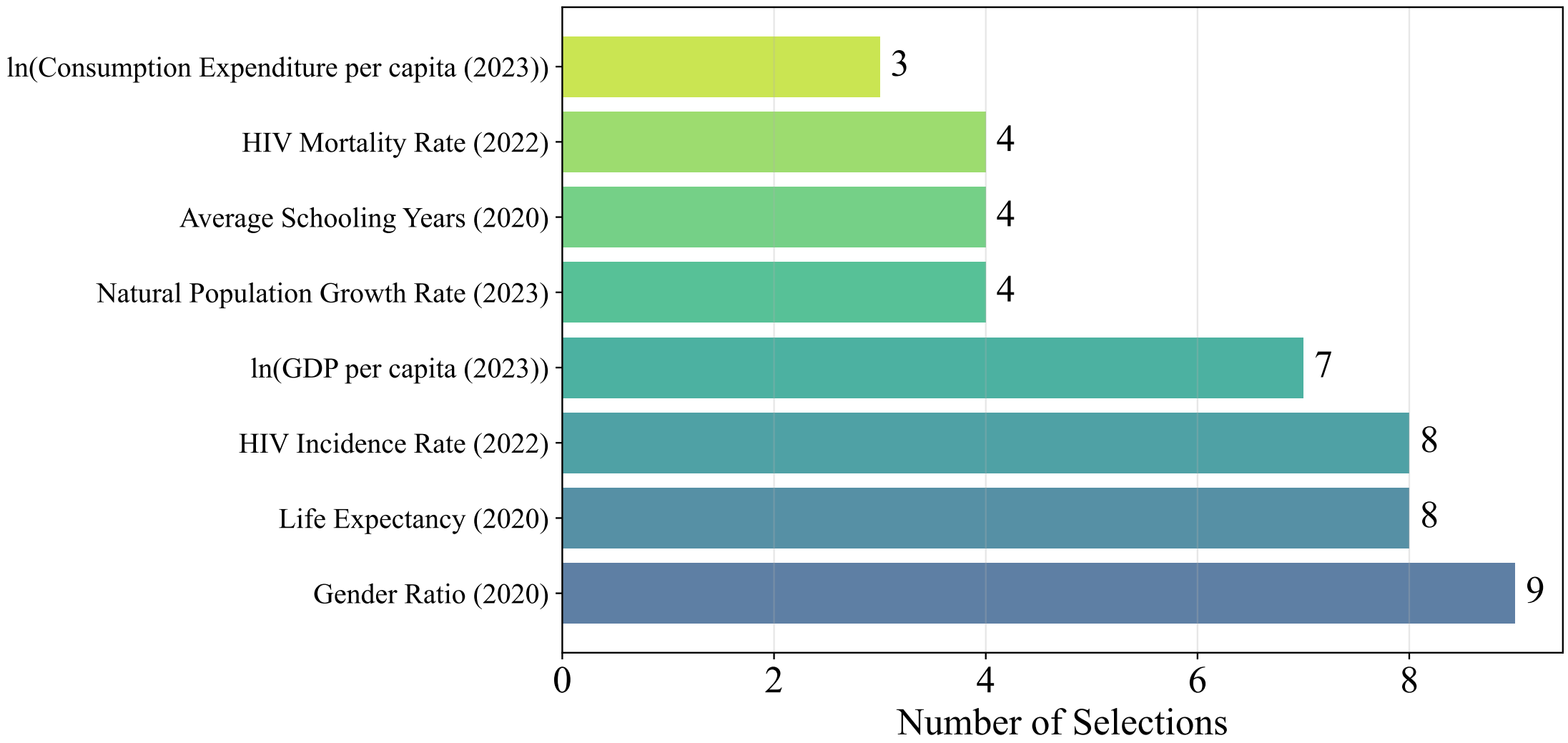

Figure A.3 Ranking of Variable Selection Frequency in LASSO Regression
