# Supplementary material for: Spatial Heterogeneity of Digital HIV/AIDS Stigma in China: Cross-Sectional Ecological Study
Source: J Med Internet Res. 2026 Oct 9;28:e93007. doi: 10.2196/93007 (PMC13652526; doi:10.2196/93007)
Supplement: Supplementary file 5 [file jmir-v28-e93007-s005.pdf]

Multimedia Appendix 4 VIF test results for the four selected variables

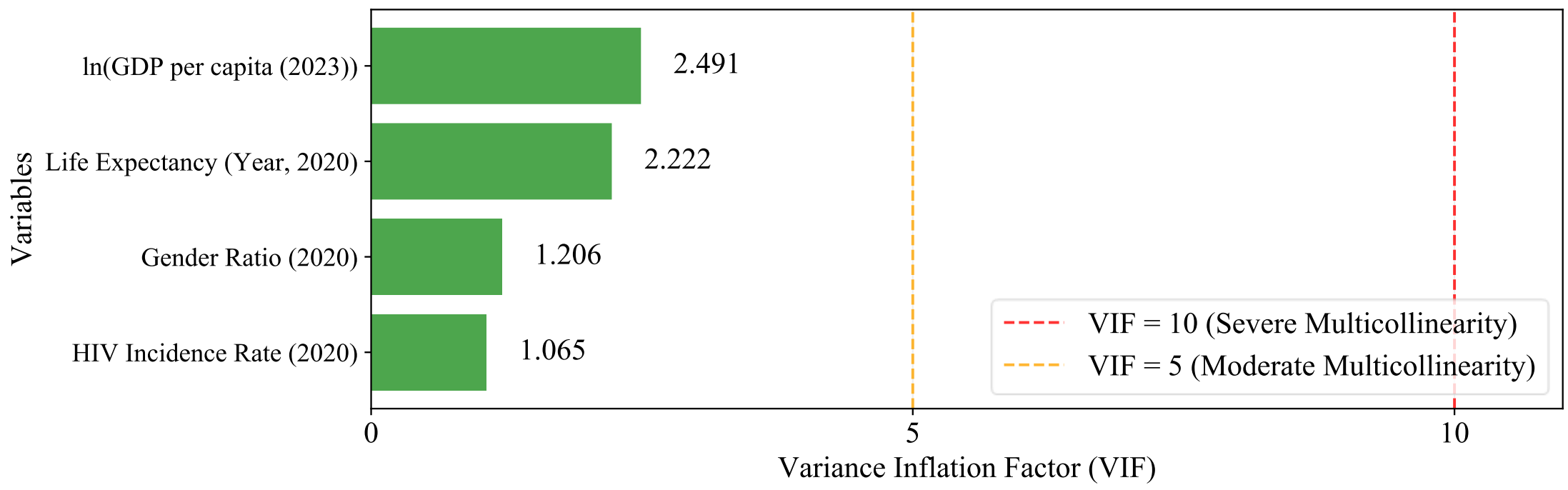

Figure A.4 VIF test results for the four selected variables
