# Supplementary material for: Spatial Heterogeneity of Digital HIV/AIDS Stigma in China: Cross-Sectional Ecological Study
Source: J Med Internet Res. 2026 Oct 9;28:e93007. doi: 10.2196/93007 (PMC13652526; doi:10.2196/93007)
Supplement: Supplementary file 6 [file jmir-v28-e93007-s006.pdf]

# Multimedia Appendix 5 Topic Index Spatial Distribution Heat Map

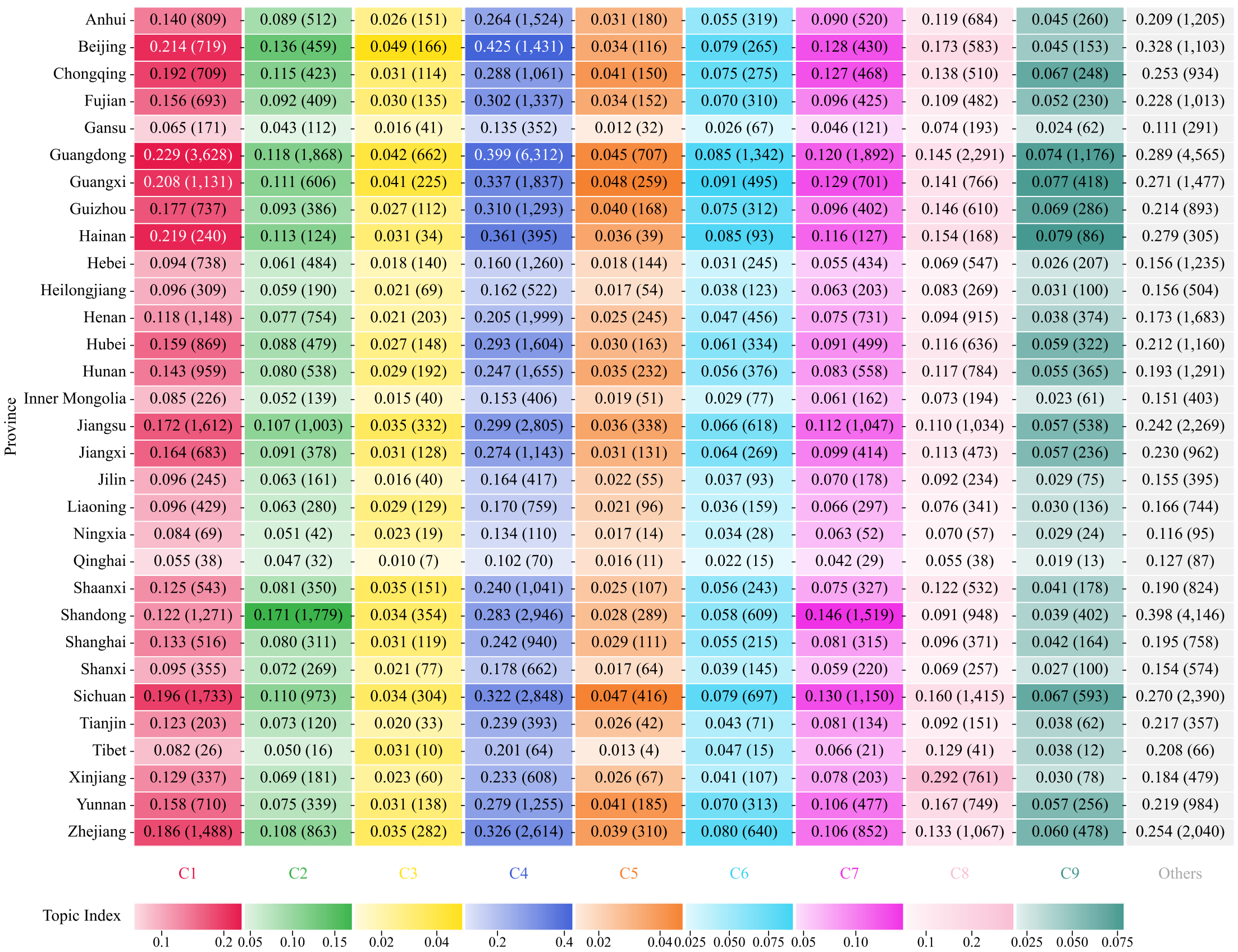

| Province | C1 | C2 | C3 | C4 | C5 | C6 | C7 | C8 | C9 | Others |
|---|---|---|---|---|---|---|---|---|---|---|
| Anhui | 0.140 (809) | 0.089 (512) | 0.026 (151) | 0.264 (1,524) | 0.031 (180) | 0.055 (319) | 0.090 (520) | 0.119 (684) | 0.045 (260) | 0.209 (1,205) |
| Beijing | 0.214 (719) | 0.136 (459) | 0.049 (166) | 0.425 (1,431) | 0.034 (116) | 0.079 (265) | 0.128 (430) | 0.173 (583) | 0.045 (153) | 0.328 (1,103) |
| Chongqing | 0.192 (709) | 0.115 (423) | 0.031 (114) | 0.288 (1,061) | 0.041 (150) | 0.075 (275) | 0.127 (468) | 0.138 (510) | 0.067 (248) | 0.253 (934) |
| Fujian | 0.156 (693) | 0.092 (409) | 0.030 (135) | 0.302 (1,337) | 0.034 (152) | 0.070 (310) | 0.096 (425) | 0.109 (482) | 0.052 (230) | 0.228 (1,013) |
| Gansu | 0.065 (171) | 0.043 (112) | 0.016 (41) | 0.135 (352) | 0.012 (32) | 0.026 (67) | 0.046 (121) | 0.074 (193) | 0.024 (62) | 0.111 (291) |
| Guangdong | 0.229 (3,628) | 0.118 (1,868) | 0.042 (662) | 0.399 (6,312) | 0.045 (707) | 0.085 (1,342) | 0.120 (1,892) | 0.145 (2,291) | 0.074 (1,176) | 0.289 (4,565) |
| Guangxi | 0.208 (1,131) | 0.111 (606) | 0.041 (225) | 0.337 (1,837) | 0.048 (259) | 0.091 (495) | 0.129 (701) | 0.141 (766) | 0.077 (418) | 0.271 (1,477) |
| Guizhou | 0.177 (737) | 0.093 (386) | 0.027 (112) | 0.310 (1,293) | 0.040 (168) | 0.075 (312) | 0.096 (402) | 0.146 (610) | 0.069 (286) | 0.214 (893) |
| Hainan | 0.219 (240) | 0.113 (124) | 0.031 (34) | 0.361 (395) | 0.036 (39) | 0.085 (93) | 0.116 (127) | 0.154 (168) | 0.079 (86) | 0.279 (305) |
| Hebei | 0.094 (738) | 0.061 (484) | 0.018 (140) | 0.160 (1,260) | 0.018 (144) | 0.031 (245) | 0.055 (434) | 0.069 (547) | 0.026 (207) | 0.156 (1,235) |
| Heilongjiang | 0.096 (309) | 0.059 (190) | 0.021 (69) | 0.162 (522) | 0.017 (54) | 0.038 (123) | 0.063 (203) | 0.083 (269) | 0.031 (100) | 0.156 (504) |
| Henan | 0.118 (1,148) | 0.077 (754) | 0.021 (203) | 0.205 (1,999) | 0.025 (245) | 0.047 (456) | 0.075 (731) | 0.094 (915) | 0.038 (374) | 0.173 (1,683) |
| Hubei | 0.159 (869) | 0.088 (479) | 0.027 (148) | 0.293 (1,604) | 0.030 (163) | 0.061 (334) | 0.091 (499) | 0.116 (636) | 0.059 (322) | 0.212 (1,160) |
| Hunan | 0.143 (959) | 0.080 (538) | 0.029 (192) | 0.247 (1,655) | 0.035 (232) | 0.056 (376) | 0.083 (558) | 0.117 (784) | 0.055 (365) | 0.193 (1,291) |
| Inner Mongolia | 0.085 (226) | 0.052 (139) | 0.015 (40) | 0.153 (406) | 0.019 (51) | 0.029 (77) | 0.061 (162) | 0.073 (194) | 0.023 (61) | 0.151 (403) |
| Jiangsu | 0.172 (1,612) | 0.107 (1,003) | 0.035 (332) | 0.299 (2,805) | 0.036 (338) | 0.066 (618) | 0.112 (1,047) | 0.110 (1,034) | 0.057 (538) | 0.242 (2,269) |
| Jiangxi | 0.164 (683) | 0.091 (378) | 0.031 (128) | 0.274 (1,143) | 0.031 (131) | 0.064 (269) | 0.099 (414) | 0.113 (473) | 0.057 (236) | 0.230 (962) |
| Jilin | 0.096 (245) | 0.063 (161) | 0.016 (40) | 0.164 (417) | 0.022 (55) | 0.037 (93) | 0.070 (178) | 0.092 (234) | 0.029 (75) | 0.155 (395) |
| Liaoning | 0.096 (429) | 0.063 (280) | 0.029 (129) | 0.170 (759) | 0.021 (96) | 0.036 (159) | 0.066 (297) | 0.076 (341) | 0.030 (136) | 0.166 (744) |
| Ningxia | 0.084 (69) | 0.051 (42) | 0.023 (19) | 0.134 (110) | 0.017 (14) | 0.034 (28) | 0.063 (52) | 0.070 (57) | 0.029 (24) | 0.116 (95) |
| Qinghai | 0.055 (38) | 0.047 (32) | 0.010 (7) | 0.102 (70) | 0.016 (11) | 0.022 (15) | 0.042 (29) | 0.055 (38) | 0.019 (13) | 0.127 (87) |
| Shaanxi | 0.125 (543) | 0.081 (350) | 0.035 (151) | 0.240 (1,041) | 0.025 (107) | 0.056 (243) | 0.075 (327) | 0.122 (532) | 0.041 (178) | 0.190 (824) |
| Shandong | 0.122 (1,271) | 0.171 (1,779) | 0.034 (354) | 0.283 (2,946) | 0.028 (289) | 0.058 (609) | 0.146 (1,519) | 0.091 (948) | 0.039 (402) | 0.398 (4,146) |
| Shanghai | 0.133 (516) | 0.080 (311) | 0.031 (119) | 0.242 (940) | 0.029 (111) | 0.055 (215) | 0.081 (315) | 0.096 (371) | 0.042 (164) | 0.195 (758) |
| Shanxi | 0.095 (355) | 0.072 (269) | 0.021 (77) | 0.178 (662) | 0.017 (64) | 0.039 (145) | 0.059 (220) | 0.069 (257) | 0.027 (100) | 0.154 (574) |
| Sichuan | 0.196 (1,733) | 0.110 (973) | 0.034 (304) | 0.322 (2,848) | 0.047 (416) | 0.079 (697) | 0.130 (1,150) | 0.160 (1,415) | 0.067 (593) | 0.270 (2,390) |
| Tianjin | 0.123 (203) | 0.073 (120) | 0.020 (33) | 0.239 (393) | 0.026 (42) | 0.043 (71) | 0.081 (134) | 0.092 (151) | 0.038 (62) | 0.217 (357) |
| Tibet | 0.082 (26) | 0.050 (16) | 0.031 (10) | 0.201 (64) | 0.013 (4) | 0.047 (15) | 0.066 (21) | 0.129 (41) | 0.038 (12) | 0.208 (66) |
| Xinjiang | 0.129 (337) | 0.069 (181) | 0.023 (60) | 0.233 (608) | 0.026 (67) | 0.041 (107) | 0.078 (203) | 0.292 (761) | 0.030 (78) | 0.184 (479) |
| Yunnan | 0.158 (710) | 0.075 (339) | 0.031 (138) | 0.279 (1,255) | 0.041 (185) | 0.070 (313) | 0.106 (477) | 0.167 (749) | 0.057 (256) | 0.219 (984) |
| Zhejiang | 0.186 (1,488) | 0.108 (863) | 0.035 (282) | 0.326 (2,614) | 0.039 (310) | 0.080 (640) | 0.106 (852) | 0.133 (1,067) | 0.060 (478) | 0.254 (2,040) |



C1: Disease Knowledge and Transmission Discussion  C2: Humor, Banter and Ironic Expression  C3: Information Sources and Credibility Discussion
C4: Moral Judgment and Attribution of Blame  C5: Personal Experience and Current Situation Sharing  C6: Risk Underestimation and Cognitive Bias
C7: Social Impact and Public Issue Discussion  C8: Support, Encouragement and Humanitarian Care  C9: Worry, Fear and Anxiety Emotions

Figure A.5 Topic Index Spatial Distribution Heat Map in Related Provinces in China
